# Selection against Heteroplasmy Explains the Evolution of Uniparental Inheritance of Mitochondria

**DOI:** 10.1371/journal.pgen.1005112

**Published:** 2015-04-16

**Authors:** Joshua R. Christie, Timothy M. Schaerf, Madeleine Beekman

**Affiliations:** 1 School of Biological Sciences, The University of Sydney, Sydney, Australia; 2 Centre for Mathematical Biology, The University of Sydney, Sydney, Australia; Brown University, UNITED STATES

## Abstract

Why are mitochondria almost always inherited from one parent during sexual reproduction? Current explanations for this evolutionary mystery include conflict avoidance between the nuclear and mitochondrial genomes, clearing of deleterious mutations, and optimization of mitochondrial-nuclear coadaptation. Mathematical models, however, fail to show that uniparental inheritance can replace biparental inheritance under any existing hypothesis. Recent empirical evidence indicates that mixing two different but normal mitochondrial haplotypes within a cell (heteroplasmy) can cause cell and organism dysfunction. Using a mathematical model, we test if selection against heteroplasmy can lead to the evolution of uniparental inheritance. When we assume selection against heteroplasmy and mutations are neither advantageous nor deleterious (neutral mutations), uniparental inheritance replaces biparental inheritance for all tested parameter values. When heteroplasmy involves mutations that are advantageous or deleterious (non-neutral mutations), uniparental inheritance can still replace biparental inheritance. We show that uniparental inheritance can evolve with or without pre-existing mating types. Finally, we show that selection against heteroplasmy can explain why some organisms deviate from strict uniparental inheritance. Thus, we suggest that selection against heteroplasmy explains the evolution of uniparental inheritance.

## Author Summary

Mitochondria contain genes that encode the machinery needed to power cells. Unlike the nuclear genome, the mitochondrial genome is typically inherited from one parent only (uniparental inheritance). The most common explanation for uniparental inheritance is the genomic conflict theory, which states that uniparental inheritance evolved to prevent the spread of ‘selfish’ mitochondria that replicate quickly but produce energy inefficiently. Current explanations have a major problem: when using realistic parameters, mathematical models cannot show that uniparental inheritance can replace biparental inheritance. Clearly, we need a new explanation that fits with standard population-genetic theory. Recent evidence suggests cells may incur a cost when they carry multiple types of mitochondria. Here we show mathematically that uniparental inheritance could have evolved to avoid the costs of maintaining multiple mitochondrial lineages within a cell. Our results explain the long-standing evolutionary mystery of uniparental inheritance and provide insight into the evolution of mating types and binary sexes. Selection against heteroplasmy also has implications for the evolution of the mitochondrial genome because new mitochondrial haplotypes always lead to heteroplasmy before becoming fixed in the population. Thus, selection against heteroplasmy may explain why mtDNA coding-genes have slower substitution rates than analogous genes within the nucleus.

## Introduction

During sexual reproduction, offspring receive two genomes: nuclear genomes from both parents and haploid cytoplasmic genomes, contained in mitochondria and chloroplasts (in plants and algae), usually from one parent. Although uniparental inheritance is nearly ubiquitous, the reasons behind its evolution remain unresolved [1, 2]. Cells contain multiple mitochondria, and the mitochondrial genome (mtDNA) encodes polypeptide subunits of the electron transport chain, which the cell uses to generate ATP via oxidative phosphorylation [2]. If mutations increase mtDNA replication rate but simultaneously decrease respiration, then increased mtDNA fitness comes at the expense of cell and organism fitness [3–5]. Nuclear and mitochondrial genomes are thus potentially in conflict. The genomic (or selfish) conflict theory argues that uniparental inheritance evolved because biparental inheritance facilitates the spread of such selfish mitochondria [1, 3–6]. Although the conflict theory has been the predominant explanation for uniparental inheritance for over three decades [3, 4], other explanations exist. A second theory suggests that uniparental inheritance facilitates the removal of deleterious mutations. Uniparental inheritance decreases variation of mtDNA within cells, but increases variation between cells, allowing purifying selection against cells with increased mutation load [1, 7]. A third hypothesis argues that because the oxidative phosphorylation pathway is composed of interacting nuclear- and mitochondrial-encoded polypeptides, uniparental inheritance optimizes mitochondrial-nuclear coadaptation by maintaining coevolved mitochondrial-nuclear combinations [1, 8]. While uniparental inheritance spreads in mathematical models of the above hypotheses [1, 5, 6], it cannot replace biparental inheritance under realistic assumptions and parameter values [1, 5]. Thus, despite decades of theoretical work, we still lack a convincing explanation for why uniparental inheritance is widespread amongst extant organisms [1, 2].

Although uniparental inheritance is the general rule in eukaryotes, there are a few exceptions. Probably the best-known exception is baker’s yeast (*Saccharomyces cerevisiae*) in which both parents contribute mitochondria to offspring [9, 10]. However, the repeated division of cells that contain two mitochondrial lineages (heteroplasmy) leads to cells that contain a single type of mitochondria (homoplasmy) [9, 10]. Another example is the male bivalve (*Mytilus*), which also inherits mitochondria from both parents. But in this case maternal and paternal mitochondria do not mix within single cells, as maternal mitochondria segregate to the soma while paternal mitochondria segregate to the gonads [11]. Thus, even when mitochondria are inherited from both parents, heteroplasmy is avoided. Recent experimental evidence suggests that this is because heteroplasmy imposes a cost on the organism. A study on mice found that the mere mixing of different, but phenotypically normal, mitochondria within a cell leads to physiological and behavioral abnormalities [12]. Could uniparental inheritance have evolved simply because carrying multiple mitochondrial types imposes a cost on the organism? Here we use a mathematical model to explore whether selection against heteroplasmy could have led to the evolution of uniparental inheritance.

### Basic model description

Our model is based on an idealized life cycle of a single-cell diploid eukaryotic organism, such as the algae *Chlamydomonas reinhardtii*. Diploid cells contain *n* mitochondria and haploid cells have *n*/2 mitochondria. All mitochondria are initially wild type but mitochondria can mutate from wild type to mutant (and vice versa). The starting population contains haploid gametes with a nuclear allele regulating biparental inheritance (*B*). Gametes are evenly split between two nuclear self-incompatible mating types (*B*_1_ and *B*_2_). In the basic model, we assume no recombination between the mitochondrial inheritance and mating type loci because these are tightly linked in many isogamous organisms [9] (later we explore recombination and no mating types). Cell types are characterized by the proportion of wild type and mutant mitochondria that they carry and their nuclear allele (haploid) or genotype (diploid).

Our life cycle has four discrete stages and is similar to the life cycles used in previous models [1, 5, 8]. Since we begin with a population of gametes, the first stage is **random mating**. Here, gametes randomly mate with the opposite mating type to produce diploid cells. Matings are controlled by the nuclear allele in gametes. In biparental inheritance (between *B*_1_ and *B*_2_ gametes), both gametes contribute mitochondria to the *B*_1_ *B*_2_ diploid cells (see later for uniparental inheritance). The second stage is **mutation.** Each mitochondrion can mutate to the other haplotype with probability *μ*. The third stage is **selection**. Here, diploid cells have a relative fitness based on the proportion of each haplotype in the cell. We assume that fitness decreases as the level of heteroplasmy increases. The fourth stage is **meiosis**, where diploid cells produce gametes that contain a single nuclear allele and *n*/2 mitochondria. As mitochondria are stochastically partitioned into gametes [9], diploid heteroplasmic cells produce gametes with varying degrees of heteroplasmy.

First, we let the population of *B*_1_ and *B*_2_ gametes reach mutation-selection equilibrium. We then simulate a mutation leading to uniparental inheritance of mitochondria by converting a small proportion (10^−2^) of *B*_1_ gametes to *U*_1_ gametes. We assume no further mutations between *B* and *U* alleles. Matings between *U*_1_ and *B*_2_ gametes result in uniparental inheritance, in which the *U*_1_ *B*_2_ cell inherits mitochondria from *U*_1_ alone. (Matings between *U*_1_ and *B*_1_ are not possible as they are the same mating type.) The population now consists of three alleles (*U*_1_, *B*_1_ and *B*_2_) and two genotypes (*U*_1_ *B*_2_ and *B*_1_ *B*_2_). The model tracks the proportion of each cell type at each stage of the life cycle. *U*_1_ spreads at the expense of *B*_1_ when uniparental inheritance is more advantageous than biparental inheritance (the frequency of *B*_2_ always remains at 0.5), and the simulation ends when the alleles reach equilibrium (see Model and S1–S6 Model for details of the model).

**Fig 1:**
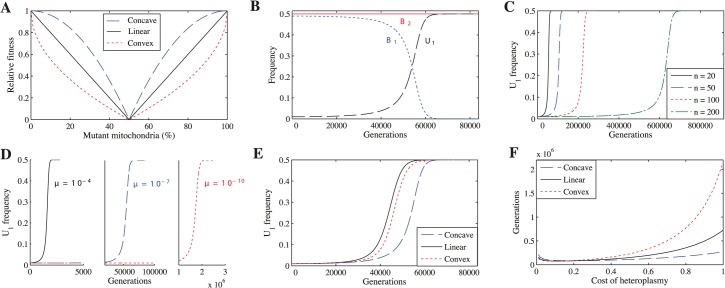
Uniparental inheritance replaces biparental inheritance for all tested parameter values. **(A)** The three fitness functions when *c*_*h*_ = 1. Unless indicated otherwise, the parameters for **B-F** are *n* = 20, *μ* = 10^−7^, *c*_*h*_ = 0.2 and concave fitness. **(B)** *U*_1_ replaces *B*_1_. **(C)** *U*_1_ takes longer to replace *B*_1_ as *n* increases. **(D)** *U*_1_ takes longer to replace *B*_1_ as *μ* decreases. **(E)** *U*_1_ replaces *B*_1_ under all three fitness functions. **(F)** Number of generations for *U*_1_ to replace *B*_1_ across a range of costs of heteroplasmy. *U*_1_ replaces *B*_1_ even if the cost of heteroplasmy is extremely low.

To explore whether a cost to heteroplasmy could have led to the evolution of uniparental inheritance, we study several scenarios. We first examine the simplest case, where mutations in mitochondria are neither advantageous nor disadvantageous (neutral mutations), but heteroplasmic cells incur a fitness cost proportional to the degree of heteroplasmy. Because no empirical data relate fitness to the degree of heteroplasmy, we consider three forms of fitness function to describe selection against heteroplasmy: concave, linear and convex (Fig 1A). For each fitness function, we vary the cost of heteroplasmy (*c*_*h*_), given by *c*_*h*_ = 1 − *h* where *h* is the fitness of the most heteroplasmic cell in the population, to see how this affects the spread of *U*_1_. We generate the concave fitness function by

$$w\left(i\right)=\{\begin{array}{cc} 1-c_{h}{\left(\frac{i}{n/2}\right)}^{2} & \text{for}\;0\leq i<n/2,\\ 1-c_{h}{\left(\frac{n-i}{n/2}\right)}^{2} & \text{for}\;n/2\leq i\leq n, \end{array}$$

the linear function by

$$w\left(i\right)=\{\begin{array}{cc} 1-c_{h}\left(\frac{i}{n/2}\right) & \text{for}0\leq i<n/2,\\ 1-c_{h}\left(\frac{n-i}{n/2}\right) & \text{for}n/2\leq i\leq n, \end{array}$$

and the convex function by

$$w\left(i\right)=\{\begin{array}{cc} 1-c_{h}\sqrt{\frac{i}{n/2}} & \text{for}\;0\leq i<n/2,\\ 1-c_{h}\sqrt{\frac{n-i}{n/2}} & \text{for}\;n/2\leq i\leq n. \end{array}$$

We also vary *μ* (mutation rate) and *n* (number of mitochondria) to ensure that our findings are robust. Second, we explore the effect of advantageous or deleterious mutations (non-neutral mutations) on the spread of *U*_1_. Third, we relax the assumption of tight linkage between mating type and inheritance loci by exploring two cases: recombination between mating types and the absence of mating types altogether. Finally, we examine whether selection against heteroplasmy can explain the rare, but nevertheless important, exceptions to uniparental inheritance. To ensure that our results generalize to more than two mitochondrial types, we developed a second model that considers three mitochondrial types (S6 Model).

## Results

### When both mitochondrial haplotypes are neutral

We find that *U*_1_ always replaces *B*_1_, resulting in complete uniparental inheritance in the population (Fig 1B). These findings are independent of the number of mitochondria per cell (Fig 1C), mutation rate (Fig 1D), fitness function (Fig 1E), and cost of heteroplasmy (Fig 1F) (see S1–S10 Tables for more parameter combinations). We find the same results when we generalize the model to three mitochondrial haplotypes (S1 Fig).

### General patterns

**Fig 2:**
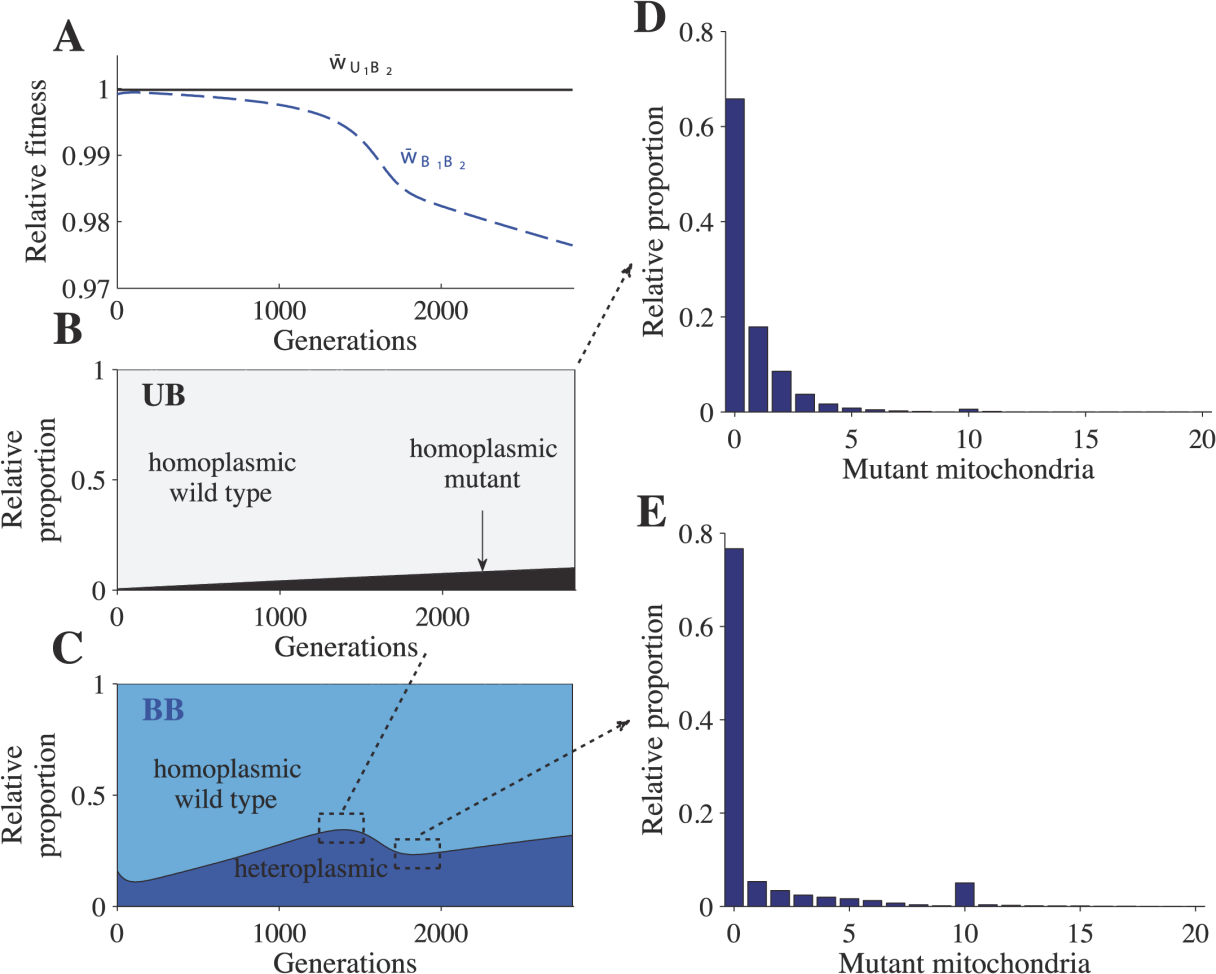
Fitness and distribution of cell types. Parameters: *n* = 20, *μ* = 10^−4^, *c*_*h*_ = 0.2 and concave fitness. *U*_1_ *B*_2_ cells appear at generation 0, which is the point at which the *B*_1_ and *B*_2_ gametes reach mutation-selection equilibrium. **(A)** Relative advantage of each genotype through time (see Model for details). For **B-E**, the relative proportion is the sum of a particular cell type divided by the sum of all cells that carry the same genotype. The heteroplasmic category includes all cells with any level of heteroplasmy. **B-C** shows the distribution of cells carrying the *U*_1_ *B*_2_ genotype **(B)** and the *B*_1_ *B*_2_ genotype **(C). D-E** show a more detailed distribution of cell types carrying the *B*_1_ *B*_2_ genotype at generation 1350 **(D)** and at generation 1820 **(E)**. The decrease in heteroplasmy in *B*_1_ *B*_2_ cells between generations 0–100 is an artifact of introducing *U*_1_ at a frequency of 0.01 (the influx of *U*_1_ gametes homoplasmic for the wild type haplotype converts some heteroplasmic *B*_1_ and *B*_2_ gametes into homoplasmic gametes, which increases the proportion of homoplasmic *B*_1_ *B*_2_ cells). From generations 1350–1820, the proportion of heteroplasmic *B*_1_ *B*_2_ cells decreases **(C)** but the level of heteroplasmy increases (compare **D** with **E**). This more than offsets the decrease in the proportion of heteroplasmic cells and ${\bar{w}}_{B_{1}B_{2}}$ continues to decrease **(A)**.

**Fig 3:**
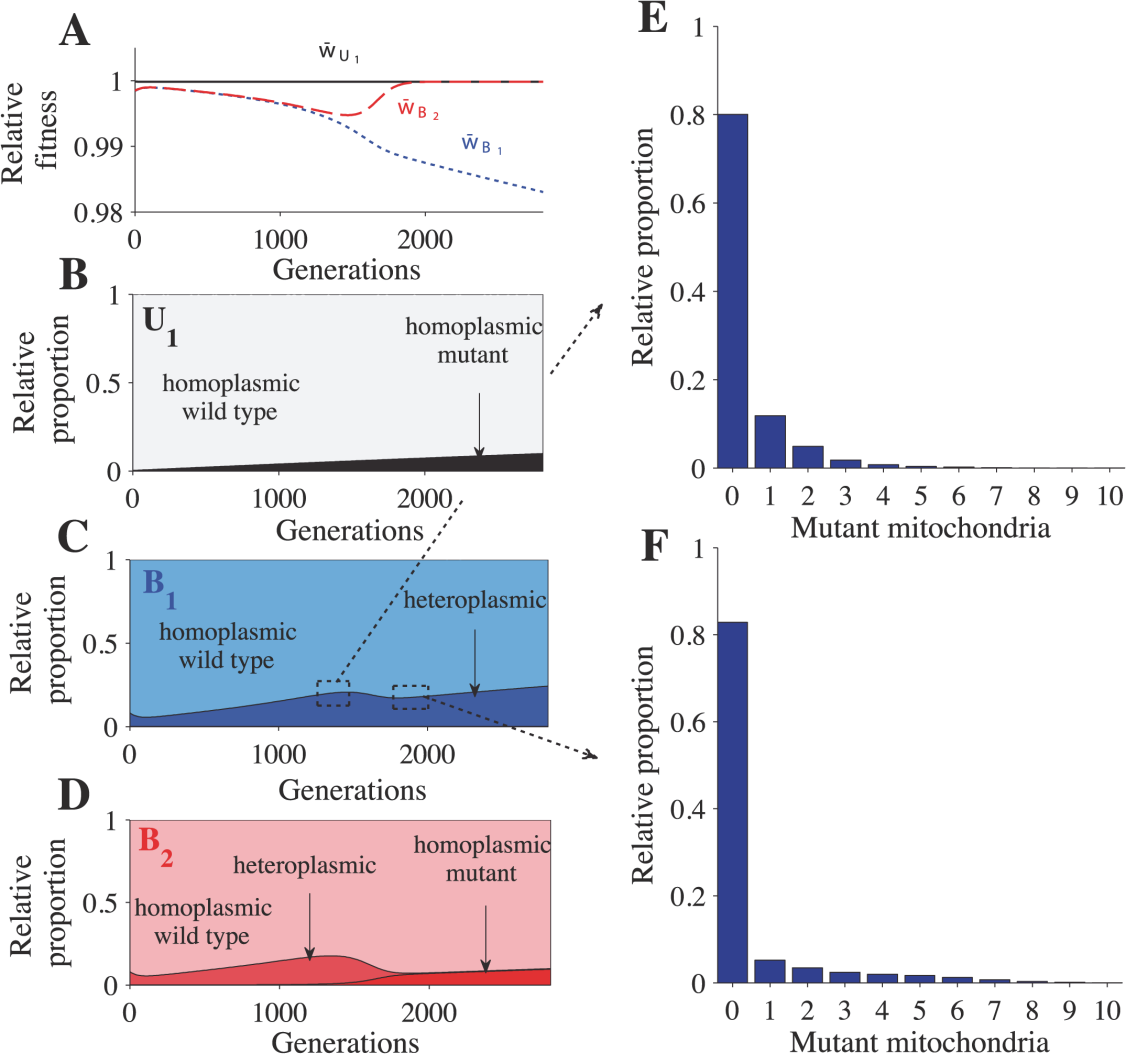
Fitness and distribution of gamete types. Parameters: *n* = 20, *μ* = 10^−4^, *c*_*h*_ = 0.2 and concave fitness. *U*_1_ gametes appear at generation 0, which is the point at which the *B*_1_ and *B*_2_ gametes reach mutation-selection equilibrium. **(A)** Relative advantage of each gamete through time (see Model for details). For **B-F**, the relative proportion is the sum of a particular gamete type (e.g. a homoplasmic wild type *U*_1_ gamete) divided by the sum of all cells carrying that allele (all gametes carrying the *U*_1_ allele). Thus, the relative proportion describes how an allele is distributed across different gamete types but it does not show their actual frequencies in the population. The heteroplasmic category combines all gametes with any level of heteroplasmy. **B-D** show the distribution of gametes carrying the *U*_1_ allele **(B)**, *B*_1_ allele **(C)** and the *B*_2_ allele **(D)**. **E-F** show a more detailed distribution of gametes carrying the *B*_1_ allele at generation 1350 **(E)** and generation 1820 **(F)**. The decrease in heteroplasmy in *B*_1_ and *B*_2_ gametes between generations 0–100 is an artifact of introducing *U*_1_ at a frequency of 0.01 (the influx of *U*_1_ gametes homoplasmic for the wild type haplotype converts some heteroplasmic *B*_1_ and *B*_2_ gametes into homoplasmic gametes). From generations 1350–1820, the proportion of heteroplasmic *B*_1_ and *B*_2_ gametes decreases **(C and D)** but the level of heteroplasmy increases (compare **E** with **F**). This more than offsets the decrease in the proportion of heteroplasmic cells and ${\bar{w}}_{B_{1}}$ continues to decrease **(A)**. Around generation 1350, *B*_2_ gametes homoplasmic for mutant mitochondria begin to appear, which causes ${\bar{w}}_{B_{2}}$ to increase and eventually converge with ${\bar{w}}_{U_{1}}$.

In our model, heteroplasmic cells are generated by mutation. During meiosis, heteroplasmic cells produce gametes with varying levels of heteroplasmy, including homoplasmic gametes. Uniparental inheritance maintains this variation created by meiosis, which leads to homoplasmic *U*_1_ *B*_2_ cells (Fig 2A–2B and S2A–S2B Fig). Mutants that arise in *U*_1_ *B*_2_ cells quickly segregate into *U*_1_ gametes that carry mutant haplotypes only (Fig 3A–3B and S3A–S3B Fig), which leads to *U*_1_ *B*_2_ cells that are homoplasmic for mutant mitochondria (Fig 2B and S2B Fig). Since we assume that mutations are neutral, cells homoplasmic for mutant mitochondria suffer no fitness costs.

*U*_1_ *B*_2_ cells carrying mutant mitochondria produce *B*_2_ gametes that also carry mutant mitochondria (Fig 3D and S3D Fig). When these *B*_2_ gametes mate with *B*_1_ gametes carrying wild type mitochondria, the resulting *B*_1_ *B*_2_ cells are highly heteroplasmic (Fig 2C–2E and S2C Fig). As *U*_1_ spreads, matings between *U*_1_ and B_2_ become more likely, increasing the level of heteroplasmy in both *B*_1_ *B*_2_ cells and in *B*_1_ and *B*_2_ gametes (Figs. 2C–2E and 3C–3F and S2C and S3C–S3D Figs.). Increased levels of heteroplasmy reduce the fitness of both *B*_1_ and *B*_2_ gametes (${\bar{w}}_{B_{1}}$, ${\bar{w}}_{B_{2}}$ in Fig 3A and S3A Fig) and *B*_1_ *B*_2_ cells (${\bar{w}}_{B_{1}B_{2}}$ in Fig 2A and S2A Fig). The difference in fitness between *B*_1_ and *B*_2_ becomes stronger (Fig 3A and S3A Fig) as more *B*_2_ gametes that carry mutant mitochondria are produced (Fig 3D and S3D Fig). As a result *U*_1_ spreads at the expense of *B*_1_.

In the above description (Figs. 2 and 3), the mutation from *B*_1_ to *U*_1_ occurred in gametes homoplasmic for wild type mitochondria. When *U*_1_ is introduced into heteroplasmic gametes, it takes fewer generations to reach equilibrium because *B*_2_ gametes homoplasmic for mutant mitochondria are produced more quickly (S4 Fig). Our results are robust to changes in the frequency at which *U*_1_ gametes are introduced (S5 Fig). For more detailed model dynamics, see S1 Text and S1–S2 Videos.

### The effect of varying parameters

*U*_1_ spreads more slowly when mutation rate (μ) is lower (Fig 1D) and number of mitochondria (*n*) is higher (Fig 1C). Reducing *μ* slows the spread of *U*_1_ because mutant mitochondria are produced more slowly, slowing the generation of *B*_2_ gametes that only carry the mutant haplotype. Increasing *n* has the same effect.

While varying the cost of heteroplasmy does not change the qualitative behavior of the model, it does affect the number of generations required for *U*_1_ to replace *B*_1_ (Fig 1F). In general, *U*_1_ spreads more quickly when the cost of heteroplasmy is low for all three fitness functions (Fig 1F). Strong selection against heteroplasmy (e.g. *c*_*h*_ = 1) slows the production of *B*_2_ gametes homoplasmic for the mutant haplotype because a transition via heteroplasmy is needed to lead to *U*_1_ *B*_2_ cells homoplasmic for mutant mitochondria. Heteroplasmy levels thus remain low in *B*_1_ *B*_2_ cells, and *U*_1_ takes longer to replace *B*_1_ (S6A and S6D Fig). At lower costs of heteroplasmy (e.g. *c*_*h*_ = 0.2), more *B*_2_ gametes that are homoplasmic for the mutant haplotype are produced and levels of heteroplasmy in *B*_1_ *B*_2_ cells increase, leading to a faster spread of *U*_1_ (S6B and S6E Fig). Although levels of heteroplasmy in *B*_1_ *B*_2_ cells increase even further as the cost of heteroplasmy approaches 0 (e.g. *c*_*h*_ = 0.01), selection against heteroplasmy is now very weak, which slows the spread of *U*_1_ compared with *c*_*h*_ = 0.2 (S6C and S6F Fig). When the number of mitochondria is higher, *U*_1_ spreads more quickly when the cost of heteroplasmy is low. This is because *B*_2_ gametes homoplasmic for mutant mitochondria are produced more slowly at higher values of *n* and strong selection against heteroplasmy compounds this problem (S7 Fig). A similar logic can be applied to understand the differences between the three fitness functions. Since heteroplasmic cells are under weaker selection when fitness is concave (followed by linear and convex respectively) (Fig 1A), the level of heteroplasmy is highest using a concave function (S8 Fig). Thus, *U*_1_ spreads more quickly using a concave function (followed by linear and convex respectively) when the cost of heteroplasmy is high because it is easier to generate heteroplasmic cells, and thus easier to generate *B*_2_ gametes homoplasmic for mutant mitochondria, when selection against heteroplasmic cells is weaker (Fig 1F and S8 Fig). As the cost of heteroplasmy decreases, the number of generations for *U*_1_ to spread under the three fitness functions converges because it becomes easier to generate *B*_2_ gametes homoplasmic for mutant mitochondria (Fig 1F).

### When mutations are deleterious

We next investigate how the *U*_1_ allele spreads when mutations are non-neutral, as is the case for most mtDNA mutations [13]. We start by assuming that mutations are deleterious so that cells carrying mutant mitochondria are more strongly selected against than cells that carry wild type mitochondria. We assume that a mutation from wild type to mutant haplotype is more common than the reverse [14]. We let the probability of a mutation from mutant to wild type haplotype be *μ*_*b*_ = *μ*/100. We vary the selection coefficient of the mutant haplotype to see how this affects the spread of the *U*_1_ allele (the fitness of a cell homoplasmic for the mutant haplotype is 1 − *s*_*d*_, where *s*_*d*_ is the selection coefficient of the mutant haplotype). Essentially there are now two fitness functions: one governing the effect of mitochondria on cell fitness (where the selection coefficient determines the magnitude of the effect) and one governing the cost of heteroplasmy. For deleterious mutations, we assume that fitness decreases as a concave function of the number of mutants, as this relationship is experimentally established [15]. We examine both concave and convex fitness functions for selection against heteroplasmy (yielding two combinations).

Again, *U*_1_ replaces *B*_1_ unless the fitness of heteroplasmic cells and the fitness of deleterious mutants are governed by a concave function and the selection coefficient is sufficiently large (S9 Fig and S11–S12 Tables). *U*_1_ generally spreads more slowly as *s*_*d*_ increases and it always spreads more slowly compared to when mutations are neutral (S11–S12 Tables). Stronger selection against mutant haplotypes leads to fewer *B*_2_ gametes homoplasmic for mutant mitochondria, which slows the spread of *U*_1_ (S10 Fig).

### When mutations are advantageous

Next we explore the effect of advantageous mutations on the spread of *U*_1_. In this case, cells that carry mutant haplotypes have an advantage over those carrying wild type haplotypes (the fitness of a cell homoplasmic for the wild type haplotype is 1 − *s*_*a*_, where *s*_*a*_ is the selection coefficient of the mutant haplotype). We account for the rarity of advantageous mutations by setting *μ*_*b*_ = 100*μ*. Because it is unknown how fitness relates to the accumulation of advantageous mtDNA mutations, we model this relationship with both a concave and convex function. As in the deleterious case, we model selection against heteroplasmy by testing both concave and convex fitness functions (giving four combinations).

*U*_1_ always replaces *B*_1_ unless mutations are highly advantageous (*s*_*a*_ = 0.1) and both the fitness of heteroplasmic cells and the fitness of advantageous mutants are governed by a concave function (S9 Fig and S13–S14 Tables). *U*_1_ spreads more quickly when *s*_*a*_ = 0.001 and *s*_*a*_ = 0.01 because *B*_2_ gametes homoplasmic for mutant haplotypes now have a fitness advantage and are produced more quickly (S10 Fig). In contrast, *U*_1_ spreads more slowly when *s*_*a*_ = 0.1 because the mutant haplotype quickly replaces the wild type as the dominant haplotype before *U*_1_ has replaced *B*_1_. Once *B*_1_ gametes carry mostly mutant haplotypes, *B*_1_ × *B*_2_ matings are less costly because they predominantly involve mutant haplotypes. We find the same patterns for non-neutral mutations when we generalize our model to three mitochondrial types (S15 Table).

### Recombination between mating type and inheritance loci

Previously, *U* × *U* matings were not possible because we assumed tight linkage between mating type and inheritance loci. But if we allow recombination to occur between these loci, *U*_1_ × *U*_2_ matings become possible. In this scenario, the number of gametes increases to four (*B*_1_, *B*_2_, *U*_1_ and *U*_2_), as does the number of genotypes (*B*_1_ *B*_2_, *U*_1_ *B*_2_, *U*_1_ *U*_2_ and *U*_2_ *B*_1_). There are three main ways in which mitochondrial inheritance could be regulated in *U*_1_ × *U*_2_ matings. (1) One *U* allele is dominant to the other, leading to uniparental inheritance; (2) each *U* allele ensures inheritance of its mitochondria, resulting in biparental inheritance; or (3) inheritance is more or less random so that some matings result in uniparental inheritance and some in biparental inheritance. We model all three cases.

**Fig 4:**
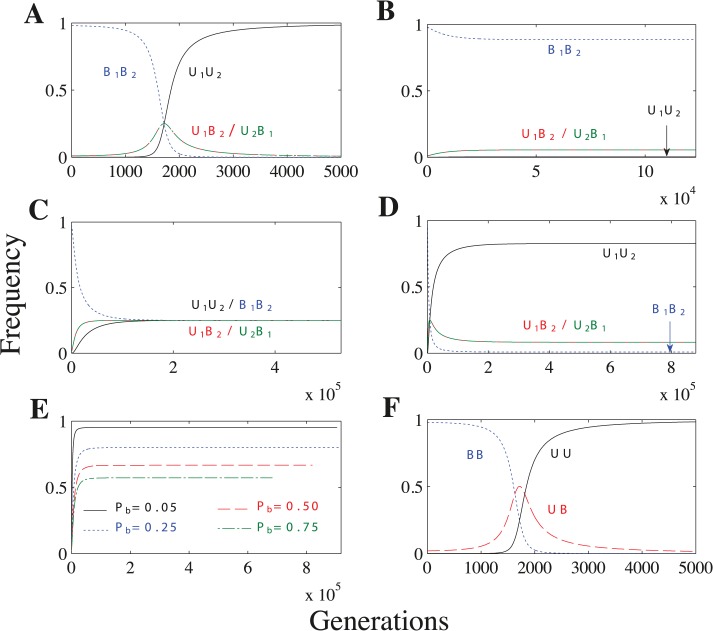
Recombination and no mating types scenarios. Parameters: *n* = 20, *μ* = 10^−4^, *c*_*h*_ = 0.2. **(A)** As the *U* allele initially spreads (generations 0–1700), the *U*_1_ *B*_2_/*U*_2_ *B*_1_ genotypes increase in frequency. But, because *U*_1_ *B*_2_ and *U*_2_ *B*_1_ cells lead to *B*_1_ *B*_2_ cells through meiosis and random mating, the *U*_1_ *U*_2_ genotype soon takes over and uniparental inheritance becomes fixed. Additional parameters: *P*_*r*_ = 0.5 and concave fitness. **(B)** Biparental inheritance dominates when *U* × *U* matings are biparental and fitness is concave. **(C)** Uniparental inheritance invades to its maximum value (0.5) when *U* × *U* matings are biparental and fitness is linear or convex. (The frequency of uniparental inheritance is the sum of *U*_1_ *U*_2_ and *U*_2_ *B*_1_.) Additional parameters: linear fitness. **(D)** *U* × *U* matings have a mixture of uniparental and biparental inheritance. Unlike in **B**, *U*_1_ *U*_2_ no longer becomes fixed because some *U* × *U* matings now have biparental inheritance and further increasing *U*_1_ *U*_2_ would only increase the overall level of biparental inheritance. Additional parameters: *P*_*b*_ = 0.1 and linear fitness. **(E)** Lines represent the frequency of uniparental inheritance in separate simulations with linear fitness and varying probabilities of biparental inheritance (*P*_*b*_) when *U* × *U* matings have a mixture of uniparental and biparental inheritance. As *P*_*b*_ increases, *U* × *U* matings are more likely to lead to biparental inheritance, which decreases the frequency of uniparental inheritance at equilibrium. **(F)** No mating types scenario under concave fitness. **F** is identical to **A** except that the frequency of *UB* in **F** is the sum of the *U*_1_ *B*_2_ and *U*_2_ *B*_1_ freqencies in **A.**

When *U*_1_ × *U*_2_ matings lead to uniparental inheritance, the *U*_1_ *U*_2_ genotype always spreads until it is fixed in the population, leading to complete uniparental inheritance (Fig 4A and S16–S18 Tables). When *U*_1_ × *U*_2_ matings lead to biparental inheritance, however, uniparental inheritance does not become fixed and the population reaches a polymorphic equilibrium (Fig 4B–4C). Under these conditions, the frequency of uniparental inheritance at equilibrium is ≤ 0.5 (S19–S21 Tables). Uniparental inheritance cannot exceed 0.5 because increasing the frequency of *U*_1_ or *U*_2_ simply increases the proportion of biparental *U*_1_ × *U*_2_ matings. The frequency of uniparental inheritance remains very low when we assume a concave fitness function (Fig 4B), but reaches its maximum (0.5) when we assume a linear or convex fitness function (Fig 4C) (see S12–S13 Figs. for an explanation).

When the probability of recombination (*P*_*r*_) is sufficiently high (10^−4^ ≤ *P*_*r*_ ≤ 0.5 in S11 Fig), the *U*_1_ *B*_2_ and *U*_2_ *B*_1_ genotypes have the same frequency at equilibrium (S11B–S11D Fig). Now uniparental inheritance is no longer associated with a single mating type but is evenly split between the two mating types (S19–S21 Tables). When *P*_*r*_ is sufficiently small (*P*_*r*_ = 10^−5^ in S11 Fig), the recombination rate is so low that the mating type and inheritance loci are essentially linked and the *U*_1_ *B*_2_ genotype becomes fixed (as in the general model) (S11A Fig).

When we assume a mixture of uniparental inheritance and biparental inheritance, we let *U*_1_ × *U*_2_ matings lead to biparental inheritance with probability *P*_*b*_ and to uniparental inheritance with probability 1 − *P*_*b*_. Lowering *P*_*b*_ increases the frequency of uniparental inheritance, and uniparental inheritance becomes fixed when *P*_*b*_ = 0 (Fig 4A and 4E). Under linear and convex fitness functions, the equilibrium always maximizes the level of uniparental inheritance (Tables S22–S23). Under concave fitness, however, uniparental inheritance is only maximized for particular values of *P*_*b*_ (roughly *P*_*b*_ ≤ 0.2 for the parameter values we considered) (S22 Table; rows 2–3). (See S5 Model for how we determine when uniparental inheritance is maximized.)

We also find that uniparental inheritance can evolve in the complete absence of mating types. The no mating types scenario differs from the recombination case in that *UB* equals the sum of *U*_1_ *B*_2_ and *U*_2_ *B*_1_ at equilibrium (Fig 4A and 4F) (see S2 Text for more details).

### Can selection against heteroplasmy explain the exceptions to uniparental inheritance?

In this section, we explore whether relaxing some of the assumptions in our general model can lead to mitochondrial inheritance patterns that resemble some of the known exceptions to uniparental inheritance. Exceptions to uniparental inheritance fall in three main categories: organisms that (1) regularly inherit mitochondria from both parents; (2) normally inherit mitochondria from one of the two parents but on occasion inherit mitochondria from both; and (3) inherit mitochondria from either or both parents.

Baker’s yeast, *Saccharomyces cerevisiae*, regularly inherits mitochondria from both parents (though uniparental inheritance also occurs), but heteroplasmy is transient because the diploid cell has only a few mitochondria [16] and divides repeatedly, which separates heteroplasmic cells into cells homoplasmic for either mitochondrial type (vegetative segregation) [9, 10]. Vegetative segregation is usually completed within twenty generations, but up to 50% of zygotes may be homoplasmic after the first division ([10] and references therein). Thus, *Saccharomyces* may restore homoplasmy as quickly as organisms that actively destroy one mitochondrial lineage [17]. Similarly, the geranium *Pelargonium zonale* often inherits cytoplasmic organelles from both parents (chloroplasts in this case). As with *Saccharomyces*, heteroplasmy is transient in *Pelargonium* because of rapid vegetative segregation of heteroplasmic cells shortly after syngamy [9]. We added mitotic divisions to our model to test whether vegetative segregation could maintain biparental inheritance under selection against heteroplasmy. When we include mitosis before selection (which assumes that vegetative segregation occurs swiftly, before selection has time to act), uniparental inheritance does not spread, provided that the number of mitochondria is low (*n* = 4) and the number of divisions is high (S24 Table; rows 7–8). Under these conditions, biparental inheritance is stable because heteroplasmic cells resulting from biparental inheritance segregate into homoplasmic cells before selection acts. If there are insufficient mitotic divisions, or if selection acts before vegetative segregation is complete, then uniparental inheritance replaces biparental inheritance, although it spreads much more slowly than when there are no mitotic divisions (S24 (rows 3–6) and S25 Tables). When there are more mitochondria per cell (e.g. *n* = 8), biparental inheritance is only stable if the number of cell divisions increases to compensate (S24 Tables; rows 9–10). Thus, biparental inheritance can be stable under selection against heteroplasmy but only under a narrow set of conditions, explaining why this form of inheritance is rare.

In other isogamous organisms, including the acellular slime molds *Physarum polycephalum* and *Didymium iridis* and the algae *Chlamydomonas reinhardtii*, mitochondria from both gametes mix before one mitochondrial lineage is destroyed post-fertilization, often by nucleases [18–20]. This mechanism is not perfect and these organisms sometimes deviate from strict uniparental inheritance [9, 18–20]. While uniparental inheritance is the norm in the slime mold *P*. *polycephalum*, sometimes both mitochondrial lineages survive, leading to varying degrees of biparental inheritance [18]. Could uniparental inheritance still spread under such conditions? Since mating types and inheritance loci are tightly linked in *Physarum* [18], we explore this question using our general model that assumes linkage. Now, *U*_1_ × *B*_2_ matings lead to biparental inheritance with probability *P*_*b*_ and to uniparental inheritance with probability 1 − *P*_*b*_. For the parameter values that we examined, the *U*_1_ *B*_2_ genotype always goes to fixation when *P*_*b*_ < 1 and the fitness function is linear or convex (S26 Table). (When fitness is concave, *P*_*b*_ must be roughly <0.05 for the *U*_1_ *B*_2_ genotype to become fixed.) Under these conditions, the frequency of biparental inheritance at equilibrium is equal to *P*_*b*_ (S26 Table). In this scenario, the level of biparental inheritance in the population simply reflects the likelihood that an individual mating results in biparental inheritance.

*Chlamydomonas reinhardtii* and *Didymium iridis* can inherit mitochondria from either or both parents [19, 20]. *Chlamydomonas* normally inherits mitochondria from the *mt* − parent and chloroplasts from the *mt* + parent, but under some circumstances it can inherit mitochondria from *mt* + and chloroplasts from *mt* − or mitochondria and chloroplasts from both [20]. *Didymium iridis* has random, biased, or dominant patterns of uniparental inheritance. Under random uniparental inheritance, either parental strain is equally likely to be the mitochondrial donor while, under biased inheritance, one strain is more likely to be the mitochondrial donor [19]. Under dominant inheritance, one strain is always the donor. *Didymium* also has low levels of biparental inheritance [19]. In this scenario, we test whether selection against heteroplasmy could lead to the evolution of a system with a mixture of uniparental inheritance (from either parent) and biparental inheritance. We assume that mating types can recombine and that *U*_1_ × *U*_2_ matings can lead to mitochondria being inherited from *U*_1_, *U*_2_ or both. Mitochondria are inherited from *U*_1_ with probability $P_{U_{1}}$, from *U*_2_ with probability $P_{U_{2}}$ and from both parents with probability *P*_*b*_ (where $P_{U_{1}}+P_{U_{2}}+P_{b}=1$). Now, uniparental inheritance comes from *U*_1_ × *B*_2_ matings, *U*_2_ × *B*_1_ matings and those *U*_1_×*U*_2_ matings with uniparental inheritance. Irrespective of the values of $P_{U_{1}}$ and $P_{U_{2}}$, we find the same results as with our earlier model in which *U*_1_×*U*_2_ matings led to a mixture of uniparental and biparental inheritance (S22–S23 Tables). This is because equilibrium depends only on the value of *P*_*b*_. (Since uniparental inheritance quickly eliminates most heteroplasmic cells, *U*_1_ *U*_2_ cells are almost entirely homoplasmic regardless of which gamete donates mitochondria.) Consequently, different probabilities of inheriting mitochondria biparentally (*P*_*b*_), from mating type 1 $\left(P_{U_{1}}\right)$ or from mating type 2 $\left(P_{U_{2}}\right)$ lead to a range of inheritance patterns that include uniparental inheritance (from both parents) and biparental inheritance (see S27 Table for some examples).

Lastly, selection against heteroplasmy provides an explanation for the cases in which mitochondria are inherited from one parent while chloroplasts are inherited from the other (e.g. in *Chlamydomonas* and pines [20, 21]). If uniparental inheritance simply evolved to maintain homoplasmy in cells, it should not matter which parent donates mitochondria or chloroplasts.

## Discussion

Our model shows that selection against heteroplasmy can lead to the fixation of uniparental inheritance in an ancestrally biparental population. We find that uniparental inheritance replaces biparental inheritance under almost all tested scenarios and parameter values. Our model also explains many of the known exceptions to strict uniparental inheritance. We show that uniparental inheritance can replace biparental inheritance whether mutations lead to neutral or non-neutral haplotypes. Relaxing our initial assumptions of pre-existing mating types and lack of recombination does not prevent uniparental inheritance from evolving. As we make no attempt to resolve the evolution of mating types within the context of mitochondrial inheritance, as others have previously attempted [1, 22], our findings thus leave open the possibility that mating types preceded uniparental inheritance, evolved as a consequence of uniparental inheritance, or evolved after uniparental inheritance.

In contrast to previous models, we show that uniparental inheritance can spread under realistic mutation rates and number of mitochondria per cell. The lowest value of *μ* that we tested (10^−10^) is eight orders of magnitude lower than required by the genomic conflict theory [1] and compares favorably with empirical mutation rates (10^−7^ to 10^−8^ per site per generation [23–25]). Both the genomic conflict and mutation clearance hypotheses require unrealistic mutation rates and number of mitochondria per cell for uniparental inheritance to replace biparental inheritance, while uniparental inheritance cannot replace biparental inheritance under any parameter values in the mitochondrial-nuclear coadaptation model [1]. The genomic conflict model requires a mutation rate of 1% per generation before uniparental inheritance can replace biparental inheritance [1]. The only known example that satisfies this assumption is the petite mutant in *Saccharomyces cerevisiae*, which is a hyper-mutable selfish mitochondrion that can spontaneously arise at a rate of 1% per generation [26]. Under this mutation rate, however, the genomic conflict model requires that cells contain at least 50 mitochondria [1], whereas most extant isogamous species, including *Saccharomyces*, contain fewer than 20 mitochondria at syngamy [16, 18]. As mutant mitochondria lack a transmission advantage over wild type mitochondria in the mutation clearance hypothesis, the mutation clearance model requires even higher mutation rates [1]. To the best of our knowledge, no extant organism satisfies the assumptions of the genomic conflict or mutation clearance hypotheses.

Why do our results differ from the findings of previous models? In the genomic conflict and mutation clearance models, wild type mitochondria mutate to selfish or deleterious mitochondria. Biparental inheritance results in cells that are heteroplasmic for wild type and mutant mtDNA, while *U*_1_ gametes mostly contain wild type mitochondria [1]. Because *U*_1_ purges *B*_2_ gametes of mutant mitochondria, *B*_1_×*B*_2_ matings involve increasingly fewer mutant mitochondria as the frequency of *U*_1_ increases [1, 5]. *U*_1_ is thus subject to negative frequency-dependent selection, and the population reaches equilibrium well before uniparental inheritance replaces biparental inheritance at realistic mutation rates [1]. The mitochondrial-nuclear coadaptation model assumes that mitochondria are well matched or poorly matched to nuclear alleles [1, 8]. Because mutation can lead to matched nuclear-mitochondrial states becoming unmatched, the effective mitochondrial mutation rate is lower in the mitochondrial-nuclear coadaptation model, which prevents uniparental inheritance from displacing biparental inheritance under any parameter values [1].

Evidence for a cost of heteroplasmy comes from a recent study that compared the effect of two mtDNA haplotypes (NZB and 129S6) in a cogenic nuclear background on the functioning of mice [12]. Mice homoplasmic for NZB or 129S6 were phenotypically normal, but NZB-129S6 heteroplasmic mice suffered from reduced activity, lowered food intake, compromised respiration, heightened stress response, and impaired cognition [12]. While the mechanism(s) behind the cost of heteroplasmy is unknown, there are a few possibilities. Heteroplasmy may disrupt cell signaling by altering production of reactive oxygen species (ROS) [27] and there are indications that heteroplasmy can increase mitochondrial ROS levels [28, 29], leading to phenotypes that differ from cells that are homoplasmic for either haplotype [29, 30]. Alternatively heteroplasmy may lead to deleterious interactions between polypeptides from different mitochondria within the same electron transport chain [12, 31]. Because chloroplasts also contain independent genomes, are involved in cellular bioenergetics, and generally show uniparental inheritance [9], our findings likely apply to both mitochondria and chloroplasts.

Although the evidence in mice is compelling [12], it is unknown whether selection against heteroplasmy is a general phenomenon in eukaryotes. While Sharpley and colleagues [12] used different mitochondrial lineages to construct heteroplasmic individuals, our model assumes that mutations accumulated within a single generation can cause mitochondrial types to become sufficiently distinct to lead to negative effects for the cell. At this stage we do not know how different mitochondrial genomes have to be for selection against heteroplasmy to apply. It could also be that there are regions of the genome in which heteroplasmic mutations have a stronger effect on fitness than others. To support or refute our model, we now need solid empirical data on a range of organisms showing the cost, if any, of heteroplasmy on organism fitness.

While we have referred to *n* as the number of mitochondria in the cell, *n* actually refers to the number of segregating units of mtDNA at syngamy. Mitochondria pack DNA into DNA-protein complexes called nucleoids, which themselves may contain multiple copies of mtDNA [32, 33]. It is currently unknown whether the segregating unit is the mtDNA molecule itself, the nucleoid, the mitochondrion or another level of mtDNA organization [33]. But as nucleoids are predominantly homoplasmic, even in heteroplasmic tissues [32, 33], the number of mitochondria may be a reasonable approximation of the number of segregating units in the cell. If the segregating unit is at a lower level of organization (e.g. the mtDNA molecule), then *n*, as used in our model, will apply to the number of segregating units not the number of mitochondria per cell (e.g. *n* = 200 would then apply to a cell with 200 segregating units, which may be a cell with far fewer than 200 mitochondria).

By assuming an infinite population size, a common assumption in studies of this kind [1, 5, 6, 8] we have ignored genetic drift, which can be a powerful force in population genetics. While it is beyond the scope of this study to formally model the effects of genetic drift on the evolution of uniparental inheritance, we can anticipate some of its effects. As the mutation leading to uniparental inheritance has a small advantage when its frequency is low, genetic drift will lead to the frequent loss of those mutations. Thus, the initial invasion of a mutation for uniparental inheritance may be largely determined by genetic drift rather than by positive selection. As the frequency of uniparental inheritance increases, however, so too does its advantage, reducing the probability that the mutation is lost to drift. The potential for rare mutations to be lost to drift is not unique to our model. The genomic conflict hypothesis requires stringent conditions for uniparental inheritance mutations to invade [6, 34]. Under this hypothesis, a mutation for uniparental inheritance must arise within a population that contains selfish mutants but in which the selfish mutant is not fixed. Otherwise, uniparental inheritance cannot become associated with non-selfish mitochondria. Any mutations leading to uniparental inheritance that arise outside of this window will have no selective advantage and will be more likely to be lost by genetic drift [6, 34].

### Conclusion

Selection against heteroplasmy has implications for the evolution of the mitochondrial genome. Because of a smaller effective population size, which is more strongly affected by genetic drift, and higher mutation rates, mtDNA should be less conserved than the nuclear genome [35, 36]. Indeed, mitochondrial transfer RNAs and synonymous sites mutate 5–50 times more frequently than comparable elements in the nuclear genome [35, 37]. Because the mitochondrial genome is effectively asexual, any deleterious mutations in the fittest haplotype cannot be rescued (except by unlikely back mutations). This effect, known as Muller's Ratchet, should eventually lead to irreparable genome meltdown [38, 39]. In stark contrast to theoretical predictions, however, mitochondrial coding genes are more conserved than analogous nuclear oxidative phosphorylation genes [36]. When mtDNA mutates, only one of the many mtDNA molecules in the cell is affected, leading to a heteroplasmic cell. Selection against heteroplasmy should reduce the probability that mtDNA mutations spread throughout the cell, which, in turn, should oppose changes to mtDNA. Thus, selection against heteroplasmy may not only explain the evolution of uniparental inheritance but also why mitochondrial coding genes have thus far managed to resist the effects of Muller's Ratchet.

## Model

Our model tracks the distribution of cell types through each stage of the life cycle across multiple generations. The redistribution of cell types is based on probability theory, but the model itself is deterministic. We assume that the population is effectively infinite and unaffected by genetic drift, as is regularly assumed in models such as ours [1, 5, 6, 8]. Consequently, the probability that a cell takes a particular state equates to the proportion of that cell type in the population. We take a similar approach to previous models [1, 5], but our model differs slightly in our treatment of mutation. Hastings does not include mutation [5], while Hadjivasiliou and colleagues treat mutation as a one-way process from wild-type to mutant mitochondria in the conflict and mutation clearance models [1]. When examining the mitochondrial-nuclear coadaptation model, however, Hadjivasiliou and colleagues allow mutation to proceed both ways as we have done here [1]. In our model, mutation is designed to capture the ability of a mitochondrial type to mutate from its current state to other haplotypes (one type in our main model and two types in our supplementary model, but an extremely large number of haplotypes in reality).

Diploid cell types are described by the vector $M^{t,\tau_{\alpha }}=\left(i,G\right)$, where *i* corresponds to the number of mutant mitochondria and takes values in {0,1…*n*}, *t* indicates the generation, and *τ*_*α*_ indicates the stage of the life cycle. If we know the number of mutant mitochondria (*i*), the number of wild type mitochondria (which we denote *j*) is fixed as *j* = *n* – *i*. *G* indicates the nuclear genotype and takes values in {*U*_1_ *B*_2_,*B*_1_ *B*_2_}. Gametes are described by the vector $M^{t,\tau_{\alpha }}=\left(p,g\right)$, where *p* is the number of mutant mitochondria and takes values in {0,1…*n* / 2} and *g* represents the nuclear allele and takes values in {*U*_1_,*B*_1_,*B*_2_}. The probability of obtaining a particular diploid cell type is written as $P\left(M^{t,\tau_{\alpha }}=(i,G)\right)$ and the probability of obtaining a particular gamete is written as $P\left(M^{t,\tau_{\alpha }}=\left(p,g\right)\right)$.

These probabilities can also be thought of as the proportion of the population with that particular cell or gamete type.

There are *n*+1 total mitochondrial states for diploid cells and *n* / 2+1 possible mitochondrial states for haploid cells. For the case in which mating type and inheritance loci are linked, the total number of diploid cell types is 2(*n*+1) while the total number of haploid cell types is 3(*n* / 2+1). We obtained numerical solutions to our model via scripts that we developed in MATLAB (version 2013b).

### Initialization

The starting population is evenly split between *B*_1_ and *B*_2_ gametes, and all gametes contain type wild type mitochondria (i.e. $P\left(M^{0,\tau_{1}}=\left(0,B_{1}\right)\right)=0.5,P\left(M^{0,\tau_{1}}=\left(0,B_{2}\right)\right)=0.5$ and $P\left(M^{0,\tau_{1}}=\left(p,g\right)\right)=0,\forall \;p>0\;\text{and}\;g=U_{1}$). We first allow this population to reach equilibrium, which we define as the point at which the proportion of cell types change by less than 10^−12^ (except when the probability that a mitochondrion mutates into another mitochondrion is 10^−10^ (*μ* = 10^−10^), in which case we define equilibrium to be a change of less than 10^−13^). We then introduce *U*_1_ gametes that are homoplasmic for wild type mitochondria by setting $P\left(M^{g_{e_{{}_{1}}},\;\tau_{1}}=\left(0,\;U_{1}\right)\right)=0.01$, where $g_{e_{1}}$ is the number of generations taken to reach the first equilibrium. To maintain the total proportion of the population at 1, we remove the corresponding proportion of cells from the *B*_1_ population $\left(i.e.\;P\left(M^{g_{e_{{}_{1}}},\;\tau_{1}}=\left(0,\;U_{1}\right)\right)=P\left(M^{g_{e_{{}_{1}}},\;\tau_{1}}=\left(0,\;U_{1}\right)\right)-0.01\right)$. In two instances, we alter the way in which *U*_1_ is introduced. In S4 Fig, we introduce *U*_1_ into the most heteroplasmic gamete with a frequency greater than 0.01, and in S5 Fig we vary the introductory frequency of *U*_1_. Our life cycle is very similar to the life cycle used by Hadjivasiliou and colleagues [1], which examined the genomic conflict, mutational clearance, and mitochondrial-nuclear coadaptation hypotheses.

### Random mating

Gametes with *n* / 2 mitochondria randomly mate with the opposite mating type to produce diploid cells containing *n* mitochondria. In effect, this is random mating in which all matings between the same mating type (i.e. *U*_1_ *U*_1_, *B*_1_ *B*_1_, *B*_2_ *B*_2_ and *U*_1_ *B*_1_) are lethal, and the only viable genotypes are *U*_1_ *B*_2_ and *B*_1_ *B*_2_.

### Biparental mating

Consider a biparental mating involving a gamete in state $M^{t,\tau_{1}}=\left(p,B_{1}\right)$, where *τ*_1_ is the gamete stage of the life cycle. For this gamete to produce a diploid cell with type $M^{t,\tau_{2}}=\left(i,B_{1}B_{2}\right)$, where *τ*_2_ is the diploid cell stage of the life cycle that precedes mutation, it must mate with a gamete of type $M^{t,\tau_{1}}=\left(i-p,B_{2}\right)$. The probability of this mating is $2P\left(M^{t,\tau_{1}}=\left(p,B_{1}\right)\right)P\left(M^{t,\tau_{1}}=\left(i-p,B_{2}\right)\right)$, where the factor of 2 accounts for the two ways in which we can choose *B*_1_ and *B*_2_ (*B*_1_ then *B*_2_ or *B*_2_ then *B*_1_). We restrict the values of *p* and *i* – *p* to biologically valid combinations. First, 0 ≤ *p* ≤ *n* / 2, as the *B*_1_ gamete cannot carry negative numbers of mutant mitochondria nor can it contain more mutant mitochondria than the total number of mitochondria in the gamete. Likewise, 0 ≤ *i* – *p* ≤ *n* / 2 for the *B*_2_ gamete, which, when rearranged, gives *i* – (*n* / 2) ≤ *p* ≤ *i*. Valid values for *p* lie in the range of intersection of these two inequalities, giving max(0,*i* – (*n* / 2)) ≤ *p* ≤ min(*n* / 2,*i*).

We can thus obtain the probability of forming any given diploid cell type after random mating with the sum,

$$P\left(M^{t,\tau_{2}}=\left(i,B_{1}B_{2}\right)\right)=2\left(\sum_{p=\text{max}\left(0,i-n/2\right)}^{\text{min}\left(n/2,i\right)}P\left(M^{t,\tau_{1}}=\left(p,B_{1}\right)\right)P\left(M^{t,\tau_{1}}=\left(i-p,B_{2}\right)\right)\right).$$

### Uniparental mating

Because uniparental matings between *U*_1_ and *B*_2_ gametes contain mitochondria from *U*_1_ alone, *U*_1_ *B*_2_ cells initially have *n* / 2 mitochondria. To restore the total complement of *n* mitochondria, we sample *n* / 2 mitochondria with replacement from the *n* / 2 mitochondria in the *U*_1_ *B*_2_ cell and add the *n* / 2 sampled mitochondria to the original set of mitochondria to form a cell with *n* mitochondria.

For a gamete with identity $M^{t,\tau_{1}}=\left(p,U_{1}\right)$ to produce a diploid cell with identity $M^{t,\tau_{2}}=\left(i,U_{1}B_{2}\right)$, it must sample *n* / 2 mitochondria containing *i* – *p* mutant mitochondria and *n* / 2 – (*i* – *p*) wild type mitochondria. The mitochondrial state of the *B*_2_ gamete is irrelevant because its mitochondria are discarded and we will refer to this cell as $M^{t,\tau_{1}}=\left(r,B_{2}\right)$.

Sampling of the *n* / 2 mitochondria follows a binomial distribution, which we denote *T*(*i* – *p*;*n* / 2,(2*p*) / *n*), where *i* – *p* refers to the number of mutant mitochondria that need to be sampled, *n* / 2 refers to the number of mitochondria being sampled, and (2*p*) / *n* is the probability of drawing a single mutant mitochondrion from a *U*_1_ *B*_2_ cell with *p* (out of *n* / 2) mutant mitochondria (where (2*p*) / *n* is obtained by rearranging *p* / (*n* / 2)).

The probability of sampling *i* – *p* mutant mitochondria (and (*n* / 2) – (*i* – *p*) wild type mitochondria) is given by

$$T\left(i-p;\frac{n}{2},\frac{2p}{n}\right)=\left(\begin{array}{l} n/2\\ i-p \end{array}\right){\left(\frac{2p}{n}\right)}^{i-p}{\left(1-\frac{2p}{n}\right)}^{\frac{n}{2}-i-p}.$$

The restrictions on *p* and *i* – *p* are the same as those in biparental mating. Since *U*_1_ will form the same initial *U*_1_ *B*_2_ cell regardless of the *B*_2_ gamete with which it mates, the probability of producing each type of *U*_1_ gamete is multiplied by the probability of selecting any *B*_2_ gamete. The probability of forming a given *U*_1_ *B*_2_ cell after random mating is determined by

$$P\left(M^{t,\tau_{2}}=\left(i,U_{1}B_{2}\right)\right)=\sum_{p=\text{max}\left(0,i-\frac{n}{2}\right)}^{\text{min}\left(\frac{n}{2},i\right)}\left(2P\left(M^{t,\tau_{1}}=\left(p,U_{1}\right)\right)\;T\left(i-p;\frac{n}{2},\frac{2p}{n}\right)\sum_{r=0}^{\frac{n}{2}}P\left(M^{t,\tau_{1}}=\left(r,B_{2}\right)\right)\right).$$

### Mutation

We denote the post-mutation states of cells as $M^{t,\;\tau_{3}}=\left(i,\;G\right)$, (where *τ*_3_ indicates the post-mutation life cycle stage). If we define the number of wild type mitochondria that mutate to mutant mitochondria to be *a* and the number of mutant mitochondria that mutate to wild type mitochondria as *b*, a post-mutation cell in state $M^{t,\;\tau_{3}}=\left(i,\;G\right)$ must be derived from a pre-mutation cell in state $M^{t,\;\tau_{2}}=\left(i-a+b,\;G\right)$ (because the pre-mutation cell gains *a* mutant mitochondria and loses *b* mutant mitochondria to form the post-mutation cell). Similarly, if the post-mutation cell has *j* wild type mitochondria, then the pre-mutation cell must have *j* + *a* – *b* wild type mitochondria, where *j* = *n* – *i*.

First, we must work out the probability that a cell mutates *a* of its wild type mitochondria to mutant mitochondria. We define *Y*(*a*;*n* – *i* + *a* – *b*,*μ*) as the probability that a pre-mutation cell has *a* mutations in its *n* – *i* + *a* – *b* wild type mitochondria given that each mitochondrion mutates with probability *μ*. The accumulation of mutations is binomially distributed such that

$$Y\left(a;n-i+a-b,\mu \right)=\left(\begin{array}{c} n-i+a-b\\ a \end{array}\right)\mu^{a}{\left(1-\mu \right)}^{n-i-b}.$$

Similarly, we define *Y*(*b*;*i* – *a* + *b*,*μ*_*b*_) to be the probability that a pre-mutation cell acquires *b* mutations in its *i* – *a* + *b* mutant mitochondria given that each mitochondrion mutates with probability *μ*_*b*_. This probability is given by

$$Y\left(b;i-a+b,\mu_{b}\right)=\left(\begin{array}{c} i-a+b\\ b \end{array}\right)\mu_{b}{}^{b}{\left(1-\mu_{b}\right)}^{i-a}.$$

For any combination of values for *a*, *b* and *i*, multiplying *Y*(*a*;*n* – *i* + *a* – *b*,*μ*) by *Y*(*b*;*i* – *a* + *b*,*μ*_*b*_) gives the probability of a particular transition from a pre-mutation cell with identity $M^{t,\;\tau_{3}}=\left(i-a+b,\;G\right)$ to a post-mutation cell with identity $M^{t,\;\tau_{3}}=\left(i,\;G\right)$. To get the overall probability that such a transition occurs, we multiply the probability of the transition by the proportion of pre-mutation cells in the population. To produce the post-mutation population, we sum all possible transitions between pre-mutation and post-mutation cells. All valid transitions must satisfy 0 ≤ *a* ≤ *i* (because the post-mutation cell cannot receive more than *i* mutant mitochondria) and 0 ≤ *b* ≤ *n* – *i* (because the post-mutation cell cannot receive more than *n* – *i* wild type mitochondria). Thus, we can determine the post-mutation population by

$$P\left(M^{t,\tau_{3}}=\left(i,G\right)\right)=\sum_{a=0}^{i}\sum_{b=0}^{n-i}Y\left(a;i-a+b,\mu \right)Y\left(b;n-i+a-b,\mu_{b}\right)P\left(M^{t,\tau_{2}}=\left(i-a+b,G\right)\right).$$

In the neutral scenario, *μ* = *μ*_*b*_ (i.e. the rate of mutation from wild type to mutant is equal to the rate of mutation from mutant to wild type).

### Selection

The relative fitness of a cell, *w*(*i*), is a measure of how likely a cell type is to survive and reproduce, and we assume that cells carrying multiple mitochondrial types have lower fitness. For the first fitness function, the relative fitness of a cell with *i* mutant mitochondria is determined according to the following piecewise concave function:

$$w(i)=\{\begin{array}{cc} 1-c_{h}{\left(\frac{i}{n/2}\right)}^{2} & \text{for}\;0\leq i<n/2,\\ 1-c_{h}{\left(\frac{n-i}{n/2}\right)}^{2} & \text{for}\;n/2\leq i\leq n, \end{array}$$ (1)

for even values of *n* and 0 ≤ *c*_*h*_ ≤ 1, where *c*_*h*_ is the cost of heteroplasmy. In this function, a cell containing *n* / 2 mutant and *n* / 2 wild type mitochondria has minimum relative fitness.

The post-selection population of each cell type is then given by:

$$P\left(M^{t,\tau_{4}}=\left(i,G\right)\right)=w\left(i\right)P\left(M^{t,\tau_{3}}=\left(i,G\right)\right).$$

We also make use of two alternative fitness functions. The first of these is the piecewise linear function:

$$w(i)=\{\begin{array}{cc} 1-c_{h}\left(\frac{i}{n/2}\right) & \text{for}\;0\leq i<n/2,\\ 1-c_{h}\left(\frac{n-i}{n/2}\right) & \text{for}\;n/2\leq i\leq n. \end{array}$$ (2)

The third fitness function is the piecewise convex function:

$$w(i)=\{\begin{array}{cc} 1-c_{h}\sqrt{\frac{i}{n/2}} & \text{for}\;0\leq i<n/2,\\ 1-c_{h}\sqrt{\frac{n-i}{n/2}} & \text{for}\;n/2\leq i\leq n. \end{array}$$ (3)

We normalize the post-selection population by

$$P\left(M^{t,\tau_{5}}=\left(i,G\right)\right)=\frac{P\left(M^{t,\tau_{4}}=\left(i,G\right)\right)}{\sigma },$$

where

$$\sigma =\sum_{i=0}^{n}P\left(M^{t,\tau_{4}}=\left(i,U_{1}B_{2}\right)\right)+P\left(M^{t,\tau_{4}}=\left(i,B_{1}B_{2}\right)\right),$$

so that the sum of the proportions of the population equals 1.

### Meiosis

The cell must first duplicate its chromosomes and double its mitochondrial complement (from *n* to 2*n*). This cell with 2*n* mitochondria then produces gametes with *n* / 2 mitochondria. Meiosis occurs in two steps. First, we sample *n* mitochondria with replacement from a cell containing *n* mitochondria and add the set of sampled mitochondria to the original set of mitochondria to form a cell containing 2*n* mitochondria (this is the same process that occurs in uniparental mating only with *n* mitochondria rather than *n* / 2 mitochondria). We let $M^{t,\tau_{6}}=\left(l,2G\right)$ represent the cell with doubled mitochondria and nuclear genotype, where *l* takes values in {0,1…2*n*} and 2*G* takes values in {*U*_1_ *U*_1_ *B*_2_ *B*_2_,*B*_1_ *B*_1_ *B*_2_ *B*_2_}.

For a cell to contain *l* mutant mitochondria after duplication of its mitochondria, it must sample *l* – *i* mutant mitochondria. We denote the probability of sampling *l* – *i* mutant mitochondria from $M^{t,\tau_{5}}=\left(i,G\right)$ as *F*(*l* – *i*;*n*,*i* / *n*). Sampling follows a binomial distribution such that

$$F\left(l-i;n,\frac{i}{n}\right)=\left(\begin{array}{c} n\\ l-i \end{array}\right){\left(\frac{i}{n}\right)}^{l-i}{\left(1-\frac{i}{n}\right)}^{n-l+i}.$$

We obtain $M^{t,\tau_{6}}=\left(l,2G\right)$ by

$$P\left(M^{t,\tau_{6}}=\left(l,2G\right)\right)=\sum_{i=\text{max}\left(0,l-n\right)}^{\text{min}\left(l,n\right)}F\left(l-i;n,\frac{i}{n}\right)P\left(M^{t,\tau_{5}}=\left(i,G\right)\right).$$

During the second step of meiosis, the cells with 2*n* mitochondria produce gametes with *n* / 2 mitochondria. Biologically, this occurs in two steps. In meiosis 1, the homologous chromosomes are pulled apart to produce two haploid cells that contain two identical nuclear alleles (sister chromatids) and *n* mitochondria. In meiosis 2, the two cells divide to produce four gametes, each with a single nuclear allele and *n* / 2 mitochondria. Since mitochondria segregate independently of nuclear alleles during cell partitioning, we model this as a single step.

We define *S*(*p*;2*n*,*l*,*n* / 2) to be the probability of obtaining *p* mutant mitochondria in *n* / 2 draws from a cell in state $M^{t,\tau_{6}}=\left(l,m,2G\right)$. Here, sampling is without replacement and follows a hypergeometric distribution, giving

$$S\left(p;2n,l,\frac{n}{2}\right)=\frac{\left(\begin{array}{l} l\\ p \end{array}\right)\left(\begin{array}{l} 2n-l\\ \frac{n}{2}-p \end{array}\right)}{\left(\begin{array}{l} 2n\\ \frac{n}{2} \end{array}\right)}.$$

Gametes produced by meiosis are represented by $M^{t+1,\tau_{1}}=\left(p,g\right)$. We determine the probability of obtaining a particular gamete using

$$\begin{array}{l} P\left(M^{t+1,\tau_{1}}=\left(p,U_{1}\right)\right)=\frac{1}{2}\left(\sum_{l=0}^{2n}S\left(p;2n,l,\frac{n}{2}\right)P\left(M^{t,\tau_{6}}=\left(l,U_{1}U_{1}B_{2}B_{2}\right)\right)\right),\\ P\left(M^{t+1,\tau_{1}}=\left(p,B_{1}\right)\right)=\frac{1}{2}\left(\sum_{l=0}^{2n}S\left(p;2n,l,\frac{n}{2}\right)P\left(M^{t,\tau_{6}}=\left(l,B_{1}B_{1}B_{2}B_{2}\right)\right)\right), \end{array}$$

and

$$\begin{array}{l} P\left(M^{t+1,\tau_{1}}=\left(p,B_{2}\right)\right)\\ =\frac{1}{2}\left(\sum_{l=0}^{2n}S\left(p;2n,l,\frac{n}{2}\right)P\left(M^{t,\tau_{6}}=\left(l,U_{1}U_{1}B_{2}B_{2}\right)\right)\right)\\ +\frac{1}{2}\left(\sum_{l=0}^{2n}S\left(p;2n,l,\frac{n}{2}\right)P\left(M^{t,\tau_{6}}=\left(l,B_{1}B_{1}B_{2}B_{2}\right)\right)\right). \end{array}$$

Factors of 1 / 2 in the above three equations take into account that half of the gametes produced from parent cells with nuclear genotype *U*_1_ *B*_2_ will carry the *U*_1_ allele and the other half will carry the *B*_2_ allele (with similar logic applied for gametes produced from parent cells with nuclear genotype *B*_1_ *B*_2_). Meiosis completes a single generation of the life cycle.

### Relative fitness of cells

The relative fitness of *U*_1_ *B*_2_ cells is given by

$${\bar{w}}_{{}_{U_{1}B_{2}}}=\frac{\sum_{i=0}^{n}P\left(M^{t,\tau_{3}}=\left(i,U_{1}B_{2}\right)\right)w\left(i\right)}{\sum_{i=0}^{n}P\left(M^{t,\tau_{3}}=\left(i,U_{1}B_{2}\right)\right)},$$

while the relative fitness of *B*_1_ *B*_2_ cells is

$${\bar{w}}_{{{}_{B_{1}B}}_{{}_{2}}}=\frac{\sum_{i=0}^{n}P\left(M^{t,\tau_{3}}=\left(i,B_{1}B_{2}\right)\right)w\left(i\right)}{\sum_{i=0}^{n}P\left(M^{t,\tau_{3}}=\left(i,B_{1}B_{2}\right)\right)}.$$

### Relative fitness of gametes

Although gametes are not subject to selection in our model, and thus do not technically have fitness values, it is informative to track gamete relative fitness throughout the simulation. We define a gamete’s relative fitness as the fitness that a diploid cell would have if it had the same mitochondrial composition as the gamete. Since gametes contain *n* / 2 mitochondria, they will have minimum fitness when they carry *n* / 4 wild type and *n* / 4 mutant mitochondria. To rescale the fitness function, we substitute *n* / 2 for *n* in the diploid cell fitness functions. For example, Equation (1) becomes

$$w_{g}(i)=\{\begin{array}{cc} 1-c_{h}{\left(\frac{i}{n/4}\right)}^{2} & \text{for}\;0\leq i<n/4,\\ 1-c_{h}{\left(\frac{\left(n/2\right)-i}{n/4}\right)}^{2} & \text{for}\;n/4\leq i\leq n/2. \end{array}$$

Once the fitness function is scaled to gametes, we can determine the relative fitness of the three gametes by

$$\begin{array}{l} {\bar{w}}_{{}_{U_{{}_{1}}}}=\frac{\sum_{i=0}^{n/2}P\left(M^{t,\tau_{1}}=\left(i,U_{1}\right)\right)w_{g}\left(i\right)}{\sum_{i=0}^{n/2}P\left(M^{t,\tau_{1}}=\left(i,U_{1}\right)\right)},\\ {\bar{w}}_{{}_{B_{{}_{1}}}}=\frac{\sum_{i=0}^{n/2}P\left(M^{t,\tau_{1}}=\left(i,B_{1}\right)\right)w_{g}\left(i\right)}{\sum_{i=0}^{n/2}P\left(M^{t,\tau_{1}}=\left(i,B_{1}\right)\right)}, \end{array}$$

and

$${\bar{w}}_{{}_{B_{{}_{2}}}}=\frac{\sum_{i=0}^{n/2}P\left(M^{t,\tau_{1}}=\left(i,B_{2}\right)\right)w_{g}\left(i\right)}{\sum_{i=0}^{n/2}P\left(M^{t,\tau_{1}}=\left(i,B_{2}\right)\right)}.$$

See S1–S6 Model for details of the other models.

## Supporting Information

S1 Fig Uniparental inheritance replaces biparental inheritance when we consider three mitochondrial types. Parameters: *n* = 20, *μ* = 10^−6^, *c*_*h*_ = 0.1 and concave fitness (unless indicated otherwise). **(A)** *U*_1_ replaces *B*_1_ leading to complete uniparental inheritance. **(B)** Number of generations to reach equilibrium for varying costs of heteroplasmy under concave and convex fitness. *U*_1_ is more advantageous when it takes fewer generations to reach equilibrium. **(C)** Number of generations to reach equilibrium for varying mutation rates. *U*_1_ replaces *B*_1_ under all tested values of *μ*. **(D)** Number of generations to reach equilibrium for different number of mitochondria per cell (as the model with three mitochondrial types is very computationally-intensive, we were unable to examine values of *n* above 40).
(EPS)

S2 Fig Fitness and distribution of cell types at a lower mutation rate. Parameters: *n* = 20, *μ* = 10^−7^, *c*_*h*_ = 0.2 and concave fitness. **(A)** Relative advantage of the two genotypes throughout time. The distribution of *U*_1_ *B*_2_ is shown in **(B)** and *B*_1_ *B*_2_ is shown in **(C)**.
(EPS)

S3 Fig Fitness and distribution of gamete types at a lower mutation rate. Parameters: *n* = 20, *μ* = 10^−7^, *c*_*h*_ = 0.2 and concave fitness. **(A)** Relative advantage of the three alleles throughout time. The distribution of *U*_1_ is shown in **(B)**, *B*_1_ is shown in **(C)** and *B*_2_ is shown in **(D)**.
(EPS)

S4 FigUniparental inheritance spreads more quickly when *U*_1_ mutates in a heteroplasmic *B*_1_ gamete compared to a homoplasmic gamete.The case in which *U*_1_ mutates into a homoplasmic cell is shown in **A-D**, while the heteroplasmic case is shown in **E-G**. We let *U*_1_ mutate in the most heteroplasmic *B*_1_ gamete that had a frequency of > 0.01 at the equilibrium between *B*_1_ and *B*_2_ (which was a gamete with two mutant mitochondria). *U*_1_ gametes appear at generation 0. The heteroplasmic *U*_1_ gametes are quickly lost (first few generations in **E** and **F**), leading to much higher levels of *U*_1_ gametes with mutant mitochondria (compare **F** with **B**). In turn, this leads to much higher levels of heteroplasmy in *B*_1_ and *B*_2_ (generations 0–450 in **G** and **H**), which results in a steeper drop in ${\bar{w}}_{B_{1}}$ and ${\bar{w}}_{B_{2}}$ (compare **E** with **A**) and a faster production of *B*_2_ gametes that carry mutant mitochondria (about generation 400 in **H** compared to 1400 in **D**). Consequently, *U*_1_ replaces *B*_1_ in about half the number of generations when it mutates in a heteroplasmic *B*_1_ gamete compared to a homoplasmic gamete.(EPS)

S5 Fig *U*_1_ replaces *B*_1_ when *U*_1_ is introduced at lower frequencies. *U*_*in*_ is the frequency of *U*_1_ when it mutates from the *B*_1_ gamete. It takes longer for *U*_1_ to replace *B*_1_ when it starts at a lower frequency. Parameters: *n* = 20, *μ* = 10^−7^, *c*_*h*_ = 0.2 and concave fitness.
(EPS)

S6 FigRelative fitness and levels of heteroplasmy for different costs of heteroplasmy.Parameters: *n* = 20, *μ* = 10^−7^ and concave fitness. (Note that the y-axis differs by two orders of magnitude between **D-F.**) Selection against heteroplasmy is strongest in (**A)** and (**D)**, which leads to very low levels of heteroplasmy in *B*_1_ *B*_2_ cells because few *B*_2_ gametes with mutant mitochondria are produced. Consequently it takes many generations before ${\bar{w}}_{B_{1}B_{2}}$ starts to drop substantially and *U*_1_ takes longer to replace *B*_1_ as a result. In (**B)** and (**E)**, selection against heteroplasmy is lower, which leads to more heteroplasmic *B*_1_ *B*_2_ cells and a faster spread of *U*_1_. While the levels of heteroplasmy rise dramatically as selection against heteroplasmy weakens further (**C** and **F**), this cannot compensate for the fact that heteroplasmic *B*_1_ *B*_2_ cells are weakly selected against. Thus, *U*_1_ takes longer to replace *B*_1_ compared to **B** and **E**.(EPS)

S7 Fig Generations for *U*_1_ to replace *B*_1_ for different numbers of mitochondria per cell and costs of heteroplasmy. *U*_1_ takes increasingly longer to replace *B*_1_ as the number of mitochondria per cell and cost of heteroplasmy increases. Parameters: *μ* = 10^−7^ and concave fitness.
(EPS)

S8 Fig Relative fitness and levels of heteroplasmy under the three fitness functions. Parameters: *n* = 20, *μ* = 10^−4^ and *c*_*h*_ = 0.2. Selection against heteroplasmy is weakest under the concave fitness function, followed by linear and convex fitness respectively (see Fig 1A). Under concave fitness **(A-D)**, this leads to higher levels of *U*_1_ gametes that carry the mutant haplotype (**B**). In turn, this leads to more *B*_2_ gametes that carry the mutant haplotype (**D**) and higher levels of heteroplasmy in *B*_1_ *B*_2_ cells (which can be seen through the high levels of heteroplasmy in the *B*_1_ gametes (**C**)). Levels of heteroplasmy in the *B*_1_ gamete are lower under linear **(E-H)** and convex **(I-L)** fitness functions because these functions select more strongly against heteroplasmic cells. *U*_1_ replaces *B*_1_ in a similar number of generations for each fitness function under these set of parameters because lower levels of heteroplasmy under linear and convex fitness is offset by stronger selection against heteroplasmic *B*_1_ *B*_2_ cells (see Fig 1F). *U*_1_ spreads at a similar rate for all three fitness functions when *c*_*h*_ = 0.2.
(EPS)

S9 Fig Non-neutral haplotypes with strong effects. Parameters: *s*_*d*_ = *s*_*a*_ = 0.1, *n* = 20, *μ* = 10^−7^ and *c*_*h*_ = 0.2. In all these cases, the accumulation of mutations is modeled using a concave fitness function. Concave/convex, as noted on the Fig, refers to the fitness function governing selection against heteroplasmy. *U*_1_ replaces *B*_1_ unless both the accumulation of mutations and selection against heteroplasmy are modeled using a concave function (black-solid and red-dashed lines). In these cases, the advantageous and deleterious scenarios converge to the same polymorphic equilibrium with a low level of uniparental inheritance. In the advantageous concave case (black-solid), mutant mitochondria quickly replace wild type mitochondria as the dominant haplotype (this corresponds to the rapid rise in *U*_1_ frequency to about 0.16). *B*_1_×*B*_2_ matings are now less costly because almost all matings involve mutant mitochondria (this stops the rapid spread of *U*_1_). At this point, the advantageous and deleterious scenarios are actually equivalent to each other (mutating from the advantageous mutant to the 'normal' wild type is the same as mutating from the 'normal' wild type to the deleterious mutant since the selection coefficients are the same in both cases). Thus, both cases converge to the same equilibrium. *U*_1_ does not replace *B*_1_ because it is more advantageous for *B*_1_ *B*_2_ cells to have low levels of heteroplasmy (but large numbers of mutant mitochondria) than it is for *U*_1_ *B*_2_ to have a low frequency of cells that are homoplasmic for the wild type haplotype (recall that *U*_1_ *B*_2_ cells quickly segregate into homoplasmic cells; thus, mutations from the advantageous mutant to wild type become segregated in homoplasmic wild type cells). This is because the mutant haplotype confers such a large advantage when *s*_*a*_ = 0.1. Contrast this with the advantageous case in which selection against heteroplasmy is convex (blue-dotted). Here, too, *U*_1_ stops its rapid spread once the mutant haplotype has replaced the wild type haplotype (*U*_1_ frequency of about 0.35), but now the *U*_1_ slowly spreads until it replaces *B*_1_. Because selection against heteroplasmy is convex in this case, which translates into stronger selection against low levels of heteroplasmy compared to concave selection, it is now less advantageous for *B*_1_ *B*_2_ cells to have low levels of heteroplasmy than it is for *U*_1_ *B*_2_ to have a low frequency of cells that are homoplasmic for the wild type haplotype. As a result, *U*_1_ slowly replaces *B*_1_.
(EPS)

S10 Fig Relative advantage and distribution of gamete types when mutations are advantageous, neutral and deleterious. In **A-D**, *U*_1_ spreads more quickly when under *s*_*a*_ = 0.001. *U*_1_ produces gametes that carry the mutant haplotype, which then rapidly spread in *U*_1_ *B*_2_ cells due to their fitness advantage (compare **B** to **F**). Because the mutant haplotype is linked to *U*_1_ (and to *B*_2_ through *U*_1_×*B*_2_ matings), *U*_1_ spreads more rapidly in this scenario. In **I-L**, *U*_1_ produces much fewer gametes that carry the mutant haplotype (compare **J** to **F**) because *U*_1_ *B*_2_ cells that only carry the mutant haplotype are more strongly selected against than *U*_1_ *B*_2_ cells that are homoplasmic for wild type mitochondria. This reduces the number of *B*_2_ gametes with mutant haplotypes (**L**), which reduces heteroplasmy in *B*_1_ *B*_2_ cells (seen in the lower level of heteroplasmy in *B*_1_ gametes (**K**)) and slows the spread of *U*_1_.
(EPS)

S11 Fig Probability of recombination does not affect equilibrium when it is above a threshold. (**A**) *P*_*r*_ is below the threshold, which leads to the fixation of the *U*_1_ *B*_2_ genotype. When *P*_*r*_ is above the threshold (**B-D**), the trajectories of the *U*_1_ *B*_2_ and *U*_2_ *B*_1_ genotypes converge. When *P*_*r*_ is above the threshold but is much lower than 0.5 (**B**), the frequency of *U*_1_ *B*_2_ is initially higher than that of *U*_2_ *B*_1_ (because the *U*_2_ gamete initially arises due to recombination between *U*_1_ and *B*_2_ gametes during *U*_1_×*B*_2_ matings). But, because there are initially more *U*_1_ *B*_2_ cells than *U*_2_ *B*_1_ cells, there are more recombination events in *U*_1_ *B*_2_ cells than in *U*_2_ *B*_1_ cells, which drives the *U*_1_:*U*_2_ ratio towards *U*_2_. The frequency of *U*_2_ continues to increase relative to *U*_1_ until *P*(*U*_1_) = *P*(*U*_2_), at which point the frequencies of *U*_1_ *B*_2_ and *U*_2_ *B*_1_ converge (**B**).
(EPS)

S12 FigUniparental inheritance is not maximized when *U*×*U* matings have biparental inheritance and fitness is concave.Additional parameters: *n* = 20, *μ* = 10^−4^, *c*_*h*_ = 0.2 and assuming no mating types. Under these conditions, the frequency of uniparental inheritance at equilibrium is 0.118. (**A**) The relative advantage of the three genotypes. **B-D** show the relative proportion of the *UB* (**B**), *BB* (**C**) and *UU* (**D**) cells types, where the heteroplasmy category includes all cells with any level of heteroplasmy. **E-F** show a more detailed distribution of the *UB* (**E**), *BB* (**F**) and *UU* (**G**) cells types at generation 80,000. **H-I** show the distribution of gamete types for the *U* (**H**) and *B* (**I**) alleles. The fitness of *UU* $\left(\bar{w_{UU}}\right)$ drops sharply in the very early stages of the simulation **(A)** because of an increase in *U* gametes homoplasmic for mutant mitochondria **(H)**. *w*_*UU*_ decreases because *U* gametes homoplasmic for mutant mitochondria mate with *U* gametes homoplasmic for wild type mitochondria, which leads to highly heteroplasmic *UU* cells. Shortly afterwards (up until about 1×10^4^ generations), *U* gametes homoplasmic for mutant mitochondria drop in frequency **(H)**. *w*_*UU*_ increases because there are now fewer *U*×*U* matings between mutant and wild type gametes. But it never reaches the level of *w*_*BB*_ **(A)** because *U* gametes homoplasmic for mutant haplotypes remain (compare **H** to **I**). Thus, although *UU* cells have a lower proportion of heteroplasmic cells, these cells have higher levels of heteroplasmy than *BB* cells (compare **F** with **G**; recall that cells with low levels of heteroplasmy are weakly selected against when fitness is concave). Because uniparental inheritance is under negative frequency-dependent selection, it does not spread to its maximum level.(EPS)

S13 Fig Uniparental inheritance is maximized when *U*×*U* matings have biparental inheritance and fitness is linear or convex. Additional parameters: *n* = 20, *μ* = 10^−4^, *c*_*h*_ = 0.2, convex fitness and assuming no mating types. (**A**) The relative advantage of the three genotypes. **B-D** show the relative proportion of the *UB* (**B**), *BB* (**C**) and *UU* (**D**) cells types, where the heteroplasmy category includes all cells with any level of heteroplasmy. **E-F** show a more detailed distribution of the *UB* (**E**), *BB* (**F**) and *UU* (**G**) cells types at generation 60,000. **H-I** show the distribution of gamete types for the *U* (**H**) and *B* (**I**) alleles. Compared to the situation under concave fitness (S12 Fig), when fitness is linear or convex a negligible amount of *U* gametes are homoplasmic for mutant mitochondria **(H)**. Consequently, there is no noticeable difference between *U*×*U* and *B*×*B* biparental inheritance matings (compare **F** to **G**) and *w*_*UU*_ converges with *w*_*BB*_ **(A)**. Because *U*×*B* matings are more advantageous than the biparental inheritance matings **(A)**, uniparental inheritance spreads to its maximum level under a linear or convex fitness function.
(EPS)

S14 Fig The concave and convex fitness functions used in the model that considers three mitochondrial types. **(A)** A three-dimensional fitness function that is similar to the two-dimensional concave function. Low levels of heteroplasmy incur a relatively small fitness cost. **(B)** A three-dimensional fitness function that is similar to the two-dimensional convex function. Low levels of heteroplasmy incur a relatively large fitness cost.
(EPS)

S1 Table General model: *n* = 20 and *μ =* 10^−4^. Generations means the number of generations to reach equilibrium. UPI frequency is the frequency of the *U*_1_ *B*_2_ genotype at equilibrium.
(PDF)

S2 Table General model: *n* = 20 and *μ =* 10^−7^. Generations means the number of generations to reach equilibrium. UPI frequency is the frequency of the *U*_1_ *B*_2_ genotype at equilibrium.
(PDF)

S3 Table General model: *n* = 20 and *μ =* 10^−10^. Generations means the number of generations to reach equilibrium. UPI frequency is the frequency of the *U*_1_ *B*_2_ genotype at equilibrium.
(PDF)

S4 Table General model: *n* = 50 and *μ =* 10^−4^. Generations means the number of generations to reach equilibrium. UPI frequency is the frequency of the *U*_1_ *B*_2_ genotype at equilibrium.
(PDF)

S5 Table General model: *n* = 50 and *μ =* 10^−7^. Generations means the number of generations to reach equilibrium. UPI frequency is the frequency of the *U*_1_ *B*_2_ genotype at equilibrium.
(PDF)

S6 Table General model: *n* = 50 and *μ =* 10^−10^. Generations means the number of generations to reach equilibrium. UPI frequency is the frequency of the *U*_1_ *B*_2_ genotype at equilibrium.
(PDF)

S7 Table General model: *n* = 100 and *μ =* 10^−4^. Generations means the number of generations to reach equilibrium. UPI frequency is the frequency of the *U*_1_ *B*_2_ genotype at equilibrium.
(PDF)

S8 Table General model: *n* = 100 and *μ =* 10^−7^. Generations means the number of generations to reach equilibrium. UPI frequency is the frequency of the *U*_1_ *B*_2_ genotype at equilibrium.
(PDF)

S9 Table General model: *n* = 100 and *μ =* 10^−10^. Generations means the number of generations to reach equilibrium. UPI frequency is the frequency of the *U*_1_ *B*_2_ genotype at equilibrium.
(PDF)

S10 Table General model: *n* = 200. Generations means the number of generations to reach equilibrium. UPI frequency is the frequency of the *U*_1_ *B*_2_ genotype at equilibrium.
(PDF)

S11 Table Deleterious model: *n* = 20 and *μ =* 10^−7^. Generations means the number of generations to reach equilibrium. UPI frequency is the frequency of the *U*_1_ *B*_2_ genotype at equilibrium. Fitness (heteroplasmy) is the fitness function governing the cost of heteroplasmy. The accumulation of deleterious mutations is modeled using a concave fitness function.
(PDF)

S12 Table Deleterious model: *n* = 100 and *μ =* 10^−7^. Generations means the number of generations to reach equilibrium. UPI frequency is the frequency of the *U*_1_ *B*_2_ genotype at equilibrium. Fitness (heteroplasmy) is the fitness function governing the cost of heteroplasmy. The accumulation of deleterious mutations is modeled using a concave fitness function.
(PDF)

S13 Table Advantageous model: *n* = 20 and *μ =* 10^−9^. Generations means the number of generations to reach equilibrium. UPI frequency is the frequency of the *U*_1_ *B*_2_ genotype at equilibrium. Fitness (heteroplasmy) is the fitness function governing the cost of heteroplasmy. Fitness (accumulation) is the fitness function that governs the accumulation of advantageous mutants.
(PDF)

S14 Table Advantageous model: *n* = 100 and *μ =* 10^−9^. Generations means the number of generations to reach equilibrium. UPI frequency is the frequency of the *U*_1_ *B*_2_ genotype at equilibrium. Fitness (heteroplasmy) is the fitness function governing the cost of heteroplasmy. Fitness (accumulation) is the fitness function that governs the accumulation of advantageous mutants.
(PDF)

S15 Table Non neutral scenario when we consider three mitochondrial types. Values represent the number of generations (×10^3^) to reach equilibrium for varying values of *s*_*a*_ (advantageous selection coefficient) and *s*_*d*_ (deleterious selection coefficient). When both haplotypes havel fitness, the population reaches equilibrium in 26(×10^3^) generations under the same set of parameters. Uniparental inheritance becomes fixed in all cases. Parameters: *n* = 20, *μ* = 10^−7^, *c*_*h*_ = 0.1 and concave fitness.
(PDF)

S16 Table Recombination and no mating types for *U*×*U* with uniparental inheritance: *n* = 20 and *μ* = 10^−4^. Generations means the number of generations to reach equilibrium. UPI frequency is the frequency of uniparental inheritance at equilibrium (*U*_1_ *U*_2_ for recombination and *UU* for no mating types). Additional parameters: *P*_*r*_ = 0.5 (for recombination).
(PDF)

S17 Table Recombination and no mating types for *U*×*U* with uniparental inheritance: *n* = 20 and *μ* = 10^−7^. Generations means the number of generations to reach equilibrium. UPI frequency is the frequency of uniparental inheritance at equilibrium (*U*_1_ *U*_2_ for recombination and *UU* for no mating types). Additional parameters: *P*_*r*_ = 0.5 (for recombination).
(PDF)

S18 Table Recombination and no mating types for *U*×*U* with uniparental inheritance *n* = 100 and *μ* = 10^−4^. Generations means the number of generations to reach equilibrium. UPI frequency is the frequency of uniparental inheritance at equilibrium (*U*_1_ *U*_2_ for recombination and *UU* for no mating types). Additional parameters: *P*_*r*_ = 0.5 (for recombination).
(PDF)

S19 Table Recombination and no mating types for *U*×*U* with biparental inheritance: *n* = 20 and *μ* = 10^−4^. UPI is maximized at 0.5 when *U*×*U* have biparental inheritance (see main text for explanation). UPI frequency (recomb.) is evenly split between the *U*_1_ *B*_2_ and *U*_2_ *B*_1_ genotypes at equilibrium, while the UPI frequency (no mating types) refers to the frequency of the *UB* genotype at equilibrium. Additional parameters: *P*_*r*_ = 0.5 (for recombination).
(PDF)

S20 Table Recombination and no mating types for *U*×*U* with biparental inheritance: *n* = 20 and *μ* = 10^−7^. UPI is maximized at 0.5 when *U*×*U* have biparental inheritance (see main text for explanation). UPI frequency (recomb.) is evenly split between the *U*_1_ *B*_2_ and *U*_2_ *B*_1_ genotypes at equilibrium, while the UPI frequency (no mating types) refers to the frequency of the *UB* genotype at equilibrium. Additional parameters: *P*_*r*_ = 0.5 (for recombination).
(PDF)

S21 Table Recombination and no mating types for *U*×*U* with biparental inheritance: *n* = 100 and *μ* = 10^−4^. UPI is maximized at 0.5 when *U*×*U* have biparental inheritance (see main text for explanation). UPI frequency (recomb.) is evenly split between the *U*_1_ *B*_2_ and *U*_2_ *B*_1_ genotypes at equilibrium, while the UPI frequency (no mating types) refers to the frequency of the *UB* genotype at equilibrium. Additional parameters: *P*_*r*_ = 0.5 (for recombination).
(PDF)

S22 Table Recombination and no mating types for *U*×*U* with mixed uniparental/biparental inheritance: *n* = 20 and *μ* = 10^−4^. UPI frequency (recomb.) is given by *P*(*U*_1_ *B*_2_) + *P*(*U*_2_ *B*_1_) + *P*(*U*_1_ *U*_2_)(1 – *P*_*b*_) (at equilibrium), while the UPI frequency (no mating types) is given by *P*(*UB*) + *P*(*UU*)(1 – *P*_*b*_) (at equilibrium). Additional parameters: *P*_*r*_ = 0.5 (for recombination). See S5 Model for how we determined whether or not uniparental inheritance was maximized.
(PDF)

S23 Table Recombination and no mating types for *U*×*U* with mixed uniparental/biparental inheritance: *n* = 100 and *μ* = 10^−4^. UPI frequency (recomb.) is given by *P*(*U*_1_ *B*_2_) + *P*(*U*_2_ *B*_1_) + *P*(*U*_1_ *U*_2_)(1 – *P*_*b*_) (at equilibrium), while the UPI frequency (no mating types) is given by *P*(*UB*) + *P*(*UU*)(1 – *P*_*b*_) (at equilibrium). Additional parameters: *P*_*r*_ = 0.5 (for recombination). See S5 Model for how we determined whether or not uniparental inheritance was maximized.
(PDF)

S24 Table Modeling *Saccharomyces*: vegetative segregation (mitosis) occurs before selection can act. Generations means the number of generations to reach equilibrium. UPI frequency is the frequency of uniparental inheritance at equilibrium. In rows 7, 8 and 10, in which there are few mitochondria, multiple mitotic divisions, and selection against heteroplasmy after mitosis, *U*_1_ has no selective advantage and does not spread beyond its introductory frequency (when *U*_1_ is introduced at a frequency of 0.01, the frequency of UPI is 0.02). Under these conditions, a mutation for uniparental inheritance could only spread via genetic drift; thus, biparental inheritance would be expected to remain stable if it were the ancestral condition. *The simulation in row 5 was stopped after 2 billion generations (before reaching equilibrium); while the spread of UPI was slowed in this simulation, it was not stopped.
(PDF)

S25 Table Modeling *Saccharomyces*: selection acts midway through vegetative segregation (mitosis). In this case, we apply selection after cells have gone through half of their mitotic divisions. After selection, we apply the second half of the mitotic divisions (e.g. in row one: 10 divisions, selection, 10 divisions).
(PDF)

S26 Table Modeling *Physarum*: *U*_1_×*B*_2_ matings have mixed uniparental/biparental inheritance. Generations means the number of generations to reach equilibrium. UPI frequency is the frequency of uniparental inheritance at equilibrium. UPI frequency is given by *P*(*U*_1_ *B*_2_)(1 – *P*_*b*_) (at equilibrium).
(PDF)

S27 TableModeling *Didymium* and *Chlamydomonas*: *U*_1_×*U*_2_ matings have a mixture of uniparental (from either parent) and biparental inheritance.We generated pseudo-random parameter values for *P*_*b*_, $P_{U_{1}}$ and $P_{U_{2}}$ using the 'twister' MATLAB rng. The rng values were normalized so that they sum to 1 because $P_{b}+P_{U_{1}}+P_{U_{2}}=1$. UPI(*U*_1_) is given by $P_{U_{1}}\left(U_{1}U_{2}\right)+U_{1}B_{2}$, UPI(*U*_2_) is given by $P_{U_{2}}\left(U_{1}U_{2}\right)+U_{2}B_{1}$ and BPI is given by *P*_*b*_(*U*_1_ *U*_2_)+*B*_1_ *B*_2_.(PDF)

S28 Table Mutation variables describing the transition between a pre- and post-mutation cell in the model with 3 mitochondrial types. (PDF)

S29 Table Fitness function parameters in the neutral scenario. Parameters: *n* = 20.
(PDF)

S30 Table Values of *ϕ* used to generate the selection coefficients for the deleterious scenario (column 1) and values of *φ* used produce the selection coefficients for the advantageous scenario (column 2) in the model with 3 mitochondrial types. (PDF)

S1 Text Detailed model dynamics. Here we describe in more detail the simulation depicted in Figs. 2 and 3.
(PDF)

S2 Text No mating types. Additional detail for the no mating types scenario.
(PDF)

S1 Model Advantageous and deleterious (non-neutral) models. (PDF)

S2 Model Mating types with recombination. (PDF)

S3 Model No mating types scenario. (PDF)

S4 Model Mitosis. (PDF)

S5 Model Frequencies of U and B alleles that maximize the level of uniparental inheritance. (PDF)

S6 Model Additional model that explores three mitochondrial types. (PDF)

S1 Video Distribution of gamete allele frequencies and relative fitness for the simulation in Fig 3. The top three panels show the distribution of the *U*_1_, *B*_1_ and *B*_2_ alleles and their respective frequencies. The middle three panels show a close-up of the top three panels. The bottom panel shows the relative fitness of the alleles. Frames were recorded every 10th generation (i.e. 10, 20, 30, etc.).
(AVI)

S2 Video Distribution of cell genotype frequencies and relative fitness for the simulation in Fig 2. The top two panels show the distribution of the *U*_1_ *B*_2_ and *B*_1_ *B*_2_ genotypes and their respective frequencies. The middle two panels show a close-up of the top two panels. The bottom panel shows the relative fitness of the genotypes. Frames were recorded every 10th generation (i.e. 10, 20, 30, etc.).
(AVI)

S1 Video Still Image (TIFF)

S2 Video Still Image (TIFF)
