# Supplementary material for: Selection against Heteroplasmy Explains the Evolution of Uniparental Inheritance of Mitochondria
Source: PLoS Genet. 2015 Apr 16;11(4):e1005112. doi: 10.1371/journal.pgen.1005112 (PMC4400020; doi:10.1371/journal.pgen.1005112)
Supplement: S1 Table — Generations means the number of generations to reach equilibrium. UPI frequency is the frequency of the U 1 B 2 genotype at equilibrium. (PDF) [file pgen.1005112.s015.pdf]

n	μ	Fitness	c_h	Generations	UPI frequency
20	10^{-4}	concave	0.01	9,559	1
20	10^{-4}	concave	0.1	3,152	1
20	10^{-4}	concave	0.2	2,822	1
20	10^{-4}	concave	0.5	3,440	1
20	10^{-4}	concave	1	6,921	1
20	10^{-4}	linear	0.01	7,266	1
20	10^{-4}	linear	0.1	2,712	1
20	10^{-4}	linear	0.2	2,708	1
20	10^{-4}	linear	0.5	4,411	1
20	10^{-4}	linear	1	14,837	1
20	10^{-4}	convex	0.01	5,973	1
20	10^{-4}	convex	0.1	2,531	1
20	10^{-4}	convex	0.2	2,841	1
20	10^{-4}	convex	0.5	6,229	1
20	10^{-4}	convex	1	35,175	1
