# Supplementary material for: Selection against Heteroplasmy Explains the Evolution of Uniparental Inheritance of Mitochondria
Source: PLoS Genet. 2015 Apr 16;11(4):e1005112. doi: 10.1371/journal.pgen.1005112 (PMC4400020; doi:10.1371/journal.pgen.1005112)
Supplement: S2 Table — Generations means the number of generations to reach equilibrium. UPI frequency is the frequency of the U 1 B 2 genotype at equilibrium. (PDF) [file pgen.1005112.s016.pdf]

| $n$ | $\mu$ | Fitness | $c_h$ | Generations | UPI frequency |
|---|---|---|---|---|---|
| 20 | $10^{-7}$ | concave | 0.01 | 213,688 | 1 |
| 20 | $10^{-7}$ | concave | 0.1 | 90,192 | 1 |
| 20 | $10^{-7}$ | concave | 0.2 | 83,812 | 1 |
| 20 | $10^{-7}$ | concave | 0.5 | 109,870 | 1 |
| 20 | $10^{-7}$ | concave | 1 | 273,173 | 1 |
| 20 | $10^{-7}$ | linear | 0.01 | 159,610 | 1 |
| 20 | $10^{-7}$ | linear | 0.1 | 76,918 | 1 |
| 20 | $10^{-7}$ | linear | 0.2 | 81,214 | 1 |
| 20 | $10^{-7}$ | linear | 0.5 | 151,245 | 1 |
| 20 | $10^{-7}$ | linear | 1 | 722,110 | 1 |
| 20 | $10^{-7}$ | convex | 0.01 | 132,535 | 1 |
| 20 | $10^{-7}$ | convex | 0.1 | 72,923 | 1 |
| 20 | $10^{-7}$ | convex | 0.2 | 87,410 | 1 |
| 20 | $10^{-7}$ | convex | 0.5 | 230,435 | 1 |
| 20 | $10^{-7}$ | convex | 1 | 2,151,217 | 1 |
