# Supplementary material for: Selection against Heteroplasmy Explains the Evolution of Uniparental Inheritance of Mitochondria
Source: PLoS Genet. 2015 Apr 16;11(4):e1005112. doi: 10.1371/journal.pgen.1005112 (PMC4400020; doi:10.1371/journal.pgen.1005112)
Supplement: S3 Table — Generations means the number of generations to reach equilibrium. UPI frequency is the frequency of the U 1 B 2 genotype at equilibrium. (PDF) [file pgen.1005112.s017.pdf]

n	μ	Fitness	c_h	Generations	UPI frequency
20	10^{-10}	concave	0.01	6,336,998	1
20	10^{-10}	concave	0.1	2,692,847	1
20	10^{-10}	concave	0.2	2,513,577	1
20	10^{-10}	concave	0.5	3,339,297	1
20	10^{-10}	concave	1	8,604,119	1
20	10^{-10}	linear	0.01	4,606,103	1
20	10^{-10}	linear	0.1	2,256,092	1
20	10^{-10}	linear	0.2	2,403,269	1
20	10^{-10}	linear	0.5	4,594,163	1
20	10^{-10}	linear	1	24,371,373	1
20	10^{-10}	convex	0.01	3,794,894	1
20	10^{-10}	convex	0.1	2,130,015	1
20	10^{-10}	convex	0.2	2,583,088	1
20	10^{-10}	convex	0.5	7,062,564	1
20	10^{-10}	convex	1	82,036,491	1
