# Supplementary material for: Selection against Heteroplasmy Explains the Evolution of Uniparental Inheritance of Mitochondria
Source: PLoS Genet. 2015 Apr 16;11(4):e1005112. doi: 10.1371/journal.pgen.1005112 (PMC4400020; doi:10.1371/journal.pgen.1005112)
Supplement: S4 Table — Generations means the number of generations to reach equilibrium. UPI frequency is the frequency of the U 1 B 2 genotype at equilibrium. (PDF) [file pgen.1005112.s018.pdf]

n	μ	Fitness	c_h	Generations	UPI frequency
50	10^{-4}	concave	0.01	9,962	1
50	10^{-4}	concave	0.1	4,407	1
50	10^{-4}	concave	0.2	4,852	1
50	10^{-4}	concave	0.5	8,975	1
50	10^{-4}	concave	1	37,751	1
50	10^{-4}	linear	0.01	7,449	1
50	10^{-4}	linear	0.1	3,998	1
50	10^{-4}	linear	0.2	5,165	1
50	10^{-4}	linear	0.5	14,322	1
50	10^{-4}	linear	1	91,882	1
50	10^{-4}	convex	0.01	5,998	1
50	10^{-4}	convex	0.1	3,986	1
50	10^{-4}	convex	0.2	6,142	1
50	10^{-4}	convex	0.5	20,662	1
50	10^{-4}	convex	1	28,866	1
