# Supplementary material for: Selection against Heteroplasmy Explains the Evolution of Uniparental Inheritance of Mitochondria
Source: PLoS Genet. 2015 Apr 16;11(4):e1005112. doi: 10.1371/journal.pgen.1005112 (PMC4400020; doi:10.1371/journal.pgen.1005112)
Supplement: S5 Table — Generations means the number of generations to reach equilibrium. UPI frequency is the frequency of the U 1 B 2 genotype at equilibrium. (PDF) [file pgen.1005112.s019.pdf]

n	μ	Fitness	c_h	Generations	UPI frequency
50	10^{-7}	concave	0.01	244,409	1
50	10^{-7}	concave	0.1	134,131	1
50	10^{-7}	concave	0.2	149,724	1
50	10^{-7}	concave	0.5	287,123	1
50	10^{-7}	concave	1	1,243,612	1
50	10^{-7}	linear	0.01	169,227	1
50	10^{-7}	linear	0.1	116,635	1
50	10^{-7}	linear	0.2	159,210	1
50	10^{-7}	linear	0.5	512,225	1
50	10^{-7}	linear	1	4,741,051	1
50	10^{-7}	convex	0.01	136,005	1
50	10^{-7}	convex	0.1	119,395	1
50	10^{-7}	convex	0.2	201,119	1
50	10^{-7}	convex	0.5	1,083,941	1
50	10^{-7}	convex	1	15,914,102	1
