# Supplementary material for: Selection against Heteroplasmy Explains the Evolution of Uniparental Inheritance of Mitochondria
Source: PLoS Genet. 2015 Apr 16;11(4):e1005112. doi: 10.1371/journal.pgen.1005112 (PMC4400020; doi:10.1371/journal.pgen.1005112)
Supplement: S6 Table — Generations means the number of generations to reach equilibrium. UPI frequency is the frequency of the U 1 B 2 genotype at equilibrium. (PDF) [file pgen.1005112.s020.pdf]

n	μ	Fitness	c_h	Generations	UPI frequency
50	10^{-10}	concave	0.01	7,424,669	1
50	10^{-10}	concave	0.1	4,046,515	1
50	10^{-10}	concave	0.2	4,520,632	1
50	10^{-10}	concave	0.5	8,745,778	1
50	10^{-10}	concave	1	40,979,076	1
50	10^{-10}	linear	0.01	4,884,210	1
50	10^{-10}	linear	0.1	3,408,579	1
50	10^{-10}	linear	0.2	4,685,580	1
50	10^{-10}	linear	0.5	15,852,680	1
50	10^{-10}	linear	1	238,657,143	1
50	10^{-10}	convex	0.01	3,878,281	1
50	10^{-10}	convex	0.1	3,459,168	1
50	10^{-10}	convex	0.2	5,897,761	1
50	10^{-10}	convex	0.5	36,072,697	1
