# Supplementary material for: Selection against Heteroplasmy Explains the Evolution of Uniparental Inheritance of Mitochondria
Source: PLoS Genet. 2015 Apr 16;11(4):e1005112. doi: 10.1371/journal.pgen.1005112 (PMC4400020; doi:10.1371/journal.pgen.1005112)
Supplement: S7 Table — Generations means the number of generations to reach equilibrium. UPI frequency is the frequency of the U 1 B 2 genotype at equilibrium. (PDF) [file pgen.1005112.s021.pdf]

n	μ	Fitness	c_h	Generations	UPI frequency
100	10^{-4}	concave	0.01	10,419	1
100	10^{-4}	concave	0.1	6,769	1
100	10^{-4}	concave	0.2	9,736	1
100	10^{-4}	concave	0.5	36,903	1
100	10^{-4}	concave	1	1,633,117	1
100	10^{-4}	linear	0.01	7,887	1
100	10^{-4}	linear	0.1	6,746	1
100	10^{-4}	linear	0.2	11,769	1
100	10^{-4}	linear	0.5	58,316	1
100	10^{-4}	linear	1	142,970	1
100	10^{-4}	convex	0.01	6,386	1
100	10^{-4}	convex	0.1	7,278	1
100	10^{-4}	convex	0.2	13,907	1
100	10^{-4}	convex	0.5	19,195	1
100	10^{-4}	convex	1	15,893	1
