# Supplementary material for: Selection against Heteroplasmy Explains the Evolution of Uniparental Inheritance of Mitochondria
Source: PLoS Genet. 2015 Apr 16;11(4):e1005112. doi: 10.1371/journal.pgen.1005112 (PMC4400020; doi:10.1371/journal.pgen.1005112)
Supplement: S8 Table — Generations means the number of generations to reach equilibrium. UPI frequency is the frequency of the U 1 B 2 genotype at equilibrium. (PDF) [file pgen.1005112.s022.pdf]

n	μ	Fitness	c_h	Generations	UPI frequency
100	10^{-7}	concave	0.01	276,437	1
100	10^{-7}	concave	0.1	213,769	1
100	10^{-7}	concave	0.2	296,028	1
100	10^{-7}	concave	0.5	903,755	1
100	10^{-7}	concave	1	8,724,257	1
100	10^{-7}	linear	0.01	185,960	1
100	10^{-7}	linear	0.1	202,757	1
100	10^{-7}	linear	0.2	373,481	1
100	10^{-7}	linear	0.5	2,170,969	1
100	10^{-7}	linear	1	42,742,860	1
100	10^{-7}	convex	0.01	149,338	1
100	10^{-7}	convex	0.1	236,540	1
100	10^{-7}	convex	0.2	584,651	1
100	10^{-7}	convex	0.5	5,688,431	1
100	10^{-7}	convex	1	10,766,453	1
