# Supplementary material for: Selection against Heteroplasmy Explains the Evolution of Uniparental Inheritance of Mitochondria
Source: PLoS Genet. 2015 Apr 16;11(4):e1005112. doi: 10.1371/journal.pgen.1005112 (PMC4400020; doi:10.1371/journal.pgen.1005112)
Supplement: S9 Table — Generations means the number of generations to reach equilibrium. UPI frequency is the frequency of the U 1 B 2 genotype at equilibrium. (PDF) [file pgen.1005112.s023.pdf]

n	μ	Fitness	c_h	Generations	UPI frequency
100	10^{-10}	concave	0.01	8,841,849	1
100	10^{-10}	concave	0.1	6,671,529	1
100	10^{-10}	concave	0.2	9,198,479	1
100	10^{-10}	concave	0.5	27,795,323	1
100	10^{-10}	concave	1	256,319,556	1
100	10^{-10}	linear	0.01	5,368,580	1
100	10^{-10}	linear	0.1	5,912,779	1
100	10^{-10}	linear	0.2	10,981,277	1
100	10^{-10}	linear	0.5	71,317,346	1
100	10^{-10}	convex	0.01	4,247,519	1
100	10^{-10}	convex	0.1	6,821,227	1
100	10^{-10}	convex	0.2	17,243,560	1
