# Supplementary material for: Selection against Heteroplasmy Explains the Evolution of Uniparental Inheritance of Mitochondria
Source: PLoS Genet. 2015 Apr 16;11(4):e1005112. doi: 10.1371/journal.pgen.1005112 (PMC4400020; doi:10.1371/journal.pgen.1005112)
Supplement: S10 Table — Generations means the number of generations to reach equilibrium. UPI frequency is the frequency of the U 1 B 2 genotype at equilibrium. (PDF) [file pgen.1005112.s024.pdf]

n	μ	Fitness	c_h	Generations	UPI frequency
200	10^{-4}	concave	0.2	31,870	1
200	10^{-4}	linear	0.2	39,882	1
200	10^{-4}	convex	0.2	16,331	1
200	10^{-7}	concave	0.2	793,592	1
200	10^{-7}	linear	0.2	1,327,599	1
200	10^{-7}	convex	0.2	2,684,316	1
200	10^{-10}	concave	0.2	24,057,028	1
