# Supplementary material for: Selection against Heteroplasmy Explains the Evolution of Uniparental Inheritance of Mitochondria
Source: PLoS Genet. 2015 Apr 16;11(4):e1005112. doi: 10.1371/journal.pgen.1005112 (PMC4400020; doi:10.1371/journal.pgen.1005112)
Supplement: S11 Table — Generations means the number of generations to reach equilibrium. UPI frequency is the frequency of the U 1 B 2 genotype at equilibrium. Fitness (heteroplasmy) is the fitness function governing the cost of heteroplasmy. The accumulation of deleterious mutations is modeled using a concave fitness function. (PDF) [file pgen.1005112.s025.pdf]

n	μ	Fitness (heteroplasmy)	c_h	s_d	Generations	UPI frequency
20	10^{-7}	concave	0.2	0.0001	338,799	1
20	10^{-7}	concave	0.2	0.001	3,047,268	1
20	10^{-7}	concave	0.2	0.01	28,737,200	1
20	10^{-7}	concave	0.2	0.1	91,748,281	**0.0923**
20	10^{-7}	convex	0.2	0.0001	357,148	1
20	10^{-7}	convex	0.2	0.001	2,912,525	1
20	10^{-7}	convex	0.2	0.01	17,605,479	1
20	10^{-7}	convex	0.2	0.1	48,894,396	1
