# Supplementary material for: Selection against Heteroplasmy Explains the Evolution of Uniparental Inheritance of Mitochondria
Source: PLoS Genet. 2015 Apr 16;11(4):e1005112. doi: 10.1371/journal.pgen.1005112 (PMC4400020; doi:10.1371/journal.pgen.1005112)
Supplement: S12 Table — Generations means the number of generations to reach equilibrium. UPI frequency is the frequency of the U 1 B 2 genotype at equilibrium. Fitness (heteroplasmy) is the fitness function governing the cost of heteroplasmy. The accumulation of deleterious mutations is modeled using a concave fitness function. (PDF) [file pgen.1005112.s026.pdf]

n	μ	Fitness (heteroplasmy)	c_h	s_d	Generations	UPI frequency
100	10^{-7}	concave	0.2	0.0001	4,621,674	1
100	10^{-7}	concave	0.2	0.001	32,631,068	**0.0106**
100	10^{-7}	concave	0.2	0.01	26,767,915	**0.0089**
100	10^{-7}	concave	0.2	0.1	25,562,026	**0.0094**
100	10^{-7}	convex	0.2	0.0001	7,955,355	1
100	10^{-7}	convex	0.2	0.001	15,640,668	1
100	10^{-7}	convex	0.2	0.01	17,411,938	1
100	10^{-7}	convex	0.2	0.1	17,902,338	1
