# Supplementary material for: Selection against Heteroplasmy Explains the Evolution of Uniparental Inheritance of Mitochondria
Source: PLoS Genet. 2015 Apr 16;11(4):e1005112. doi: 10.1371/journal.pgen.1005112 (PMC4400020; doi:10.1371/journal.pgen.1005112)
Supplement: S13 Table — Generations means the number of generations to reach equilibrium. UPI frequency is the frequency of the U 1 B 2 genotype at equilibrium. Fitness (heteroplasmy) is the fitness function governing the cost of heteroplasmy. Fitness (accumulation) is the fitness function that governs the accumulation of advantageous mutants. (PDF) [file pgen.1005112.s027.pdf]

n	μ	Fitness (heteroplasmy)	Fitness (accumulation)	c_h	s_a	Generations	UPI frequency
20	10^{-9}	concave	concave	0.2	0.0001	82,264	1
20	10^{-9}	concave	concave	0.2	0.001	12,957	1
20	10^{-9}	concave	concave	0.2	0.01	2,481	1
20	10^{-9}	concave	concave	0.2	0.1	110,066,931	**0.0924**
20	10^{-9}	convex	concave	0.2	0.0001	83,404	1
20	10^{-9}	convex	concave	0.2	0.001	13,038	1
20	10^{-9}	convex	concave	0.2	0.01	1,837	1
20	10^{-9}	convex	concave	0.2	0.1	40,285,032	1
20	10^{-9}	concave	convex	0.2	0.0001	82,261	1
20	10^{-9}	concave	convex	0.2	0.001	12,955	1
20	10^{-9}	concave	convex	0.2	0.01	2,374	1
20	10^{-9}	concave	convex	0.2	0.1	92,070,225	1
20	10^{-9}	convex	convex	0.2	0.0001	83,407	1
20	10^{-9}	convex	convex	0.2	0.001	13,040	1
20	10^{-9}	convex	convex	0.2	0.01	1,836	1
20	10^{-9}	convex	convex	0.2	0.1	34,902,696	1
