# Supplementary material for: Selection against Heteroplasmy Explains the Evolution of Uniparental Inheritance of Mitochondria
Source: PLoS Genet. 2015 Apr 16;11(4):e1005112. doi: 10.1371/journal.pgen.1005112 (PMC4400020; doi:10.1371/journal.pgen.1005112)
Supplement: S14 Table — Generations means the number of generations to reach equilibrium. UPI frequency is the frequency of the U 1 B 2 genotype at equilibrium. Fitness (heteroplasmy) is the fitness function governing the cost of heteroplasmy. Fitness (accumulation) is the fitness function that governs the accumulation of advantageous mutants. (PDF) [file pgen.1005112.s028.pdf]

n	μ	Fitness (heteroplasmy)	Fitness (accumulation)	c_h	s_a	Generations	UPI frequency
100	10^{-9}	concave	concave	0.2	0.0001	107,601	1
100	10^{-9}	concave	concave	0.2	0.001	15,401	1
100	10^{-9}	concave	concave	0.2	0.01	2,826	1
100	10^{-9}	concave	concave	0.2	0.1	38,526,085	**0.0094**
100	10^{-9}	convex	concave	0.2	0.0001	123,995	1
100	10^{-9}	convex	concave	0.2	0.001	17,024	1
100	10^{-9}	convex	concave	0.2	0.01	2,215	1
100	10^{-9}	convex	concave	0.2	0.1	15,013,754	1
100	10^{-9}	concave	convex	0.2	0.0001	107,596	1
100	10^{-9}	concave	convex	0.2	0.001	15,402	1
100	10^{-9}	concave	convex	0.2	0.01	2,711	1
100	10^{-9}	concave	convex	0.2	0.1	39,494,905	1
100	10^{-9}	convex	convex	0.2	0.0001	124,004	1
100	10^{-9}	convex	convex	0.2	0.001	17,032	1
100	10^{-9}	convex	convex	0.2	0.01	2,222	1
100	10^{-9}	convex	convex	0.2	0.1	12,530,369	1
