# Supplementary material for: Selection against Heteroplasmy Explains the Evolution of Uniparental Inheritance of Mitochondria
Source: PLoS Genet. 2015 Apr 16;11(4):e1005112. doi: 10.1371/journal.pgen.1005112 (PMC4400020; doi:10.1371/journal.pgen.1005112)
Supplement: S15 Table — Values represent the number of generations (×103) to reach equilibrium for varying values of s a (advantageous selection coefficient) and s d (deleterious selection coefficient). When both haplotypes havel fitness, the population reaches equilibrium in 26(×103) generations under the same set of parameters. Uniparental inheritance becomes fixed in all cases. Parameters: n = 20, μ = 10−7, c h = 0.1 and concave fitness. (PDF) [file pgen.1005112.s029.pdf]

	$s_d = s_a = 10^{-2}$	$s_d = s_a = 10^{-3}$	$s_d = s_a = 10^{-4}$	$s_d = s_a = 10^{-5}$
Deleterious	2428	282	41	27
Advantageous	725	11	26	26
