# Supplementary material for: Selection against Heteroplasmy Explains the Evolution of Uniparental Inheritance of Mitochondria
Source: PLoS Genet. 2015 Apr 16;11(4):e1005112. doi: 10.1371/journal.pgen.1005112 (PMC4400020; doi:10.1371/journal.pgen.1005112)
Supplement: S16 Table — Generations means the number of generations to reach equilibrium. UPI frequency is the frequency of uniparental inheritance at equilibrium (U 1 U 2 for recombination and UU for no mating types). Additional parameters: P r = 0.5 (for recombination). (PDF) [file pgen.1005112.s030.pdf]

n	μ	Fitness	c_h	Generations (recomb.)	Generations (no mating types)	UPI frequency (recomb.)	UPI frequency (no mating types)
20	10^{-4}	concave	0.01	7,082,386	10,013,235	1	1
20	10^{-4}	linear	0.01	7,075,847	10,004,657	1	1
20	10^{-4}	convex	0.01	7,071,186	9,998,502	1	1
20	10^{-4}	concave	0.5	1,020,973	1,435,174	1	1
20	10^{-4}	linear	0.5	1,054,516	1,468,280	1	1
20	10^{-4}	convex	0.5	1,180,731	1,575,213	1	1
