# Supplementary material for: Selection against Heteroplasmy Explains the Evolution of Uniparental Inheritance of Mitochondria
Source: PLoS Genet. 2015 Apr 16;11(4):e1005112. doi: 10.1371/journal.pgen.1005112 (PMC4400020; doi:10.1371/journal.pgen.1005112)
Supplement: S17 Table — Generations means the number of generations to reach equilibrium. UPI frequency is the frequency of uniparental inheritance at equilibrium (U 1 U 2 for recombination and UU for no mating types). Additional parameters: P r = 0.5 (for recombination). (PDF) [file pgen.1005112.s031.pdf]

n	μ	Fitness	c_h	Generations (recomb.)	Generations (no mating types)	UPI frequency (recomb.)	UPI frequency (no mating types)
20	10^{-7}	concave	0.01	10,715,687	13,711,563	1	1
20	10^{-7}	linear	0.01	10,801,711	13,815,038	1	1
20	10^{-7}	convex	0.01	10,887,685	13,918,429	1	1
20	10^{-7}	concave	0.5	5,516,194	6,968,570	1	1
20	10^{-7}	linear	0.5	7,605,897	9,586,868	1	1
20	10^{-7}	convex	0.5	10,515,076	13,250,420	1	1
