# Supplementary material for: Selection against Heteroplasmy Explains the Evolution of Uniparental Inheritance of Mitochondria
Source: PLoS Genet. 2015 Apr 16;11(4):e1005112. doi: 10.1371/journal.pgen.1005112 (PMC4400020; doi:10.1371/journal.pgen.1005112)
Supplement: S19 Table — UPI is maximized at 0.5 when U×U have biparental inheritance (see main text for explanation). UPI frequency (recomb.) is evenly split between the U 1 B 2 and U 2 B 1 genotypes at equilibrium, while the UPI frequency (no mating types) refers to the frequency of the UB genotype at equilibrium. Additional parameters: P r = 0.5 (for recombination). (PDF) [file pgen.1005112.s033.pdf]

n	μ	Fitness	c_h	Generations (recomb.)	Generations (no mating types)	UPI frequency (recomb.)	UPI frequency (no mating types)	UPI maximized?
20	10^{-4}	concave	0.01	138,240	144,734	0.1652	0.1652	NO
20	10^{-4}	linear	0.01	655,218	686,486	0.5	0.5	YES
20	10^{-4}	convex	0.01	110,387	114,724	0.5	0.5	YES
20	10^{-4}	concave	0.5	116,062	121,328	0.1299	0.1299	NO
20	10^{-4}	linear	0.5	357,413	373,497	0.5	0.5	YES
20	10^{-4}	convex	0.5	92,255	95,749	0.5	0.5	YES
