# Supplementary material for: Selection against Heteroplasmy Explains the Evolution of Uniparental Inheritance of Mitochondria
Source: PLoS Genet. 2015 Apr 16;11(4):e1005112. doi: 10.1371/journal.pgen.1005112 (PMC4400020; doi:10.1371/journal.pgen.1005112)
Supplement: S20 Table — UPI is maximized at 0.5 when U×U have biparental inheritance (see main text for explanation). UPI frequency (recomb.) is evenly split between the U 1 B 2 and U 2 B 1 genotypes at equilibrium, while the UPI frequency (no mating types) refers to the frequency of the UB genotype at equilibrium. Additional parameters: P r = 0.5 (for recombination). (PDF) [file pgen.1005112.s034.pdf]

n	μ	Fitness	c_h	Generations (recomb.)	Generations (no mating types)	UPI frequency (recomb.)	UPI frequency (no mating types)	UPI maximized?
20	10^{-7}	concave	0.01	74,554,279	81,261,516	0.1047	0.1047	NO
20	10^{-7}	linear	0.01	382,112,603	417,762,035	0.5	0.5	YES
20	10^{-7}	convex	0.01	63,571,592	67,634,661	0.5	0.5	YES
20	10^{-7}	concave	0.5	64,183,540	69,441,956	0.1274	0.1274	NO
20	10^{-7}	linear	0.5	197,181,121	213,258,885	0.5	0.5	YES
20	10^{-7}	convex	0.5	57,399,784	60,887,876	0.5	0.5	YES
