# Supplementary material for: Selection against Heteroplasmy Explains the Evolution of Uniparental Inheritance of Mitochondria
Source: PLoS Genet. 2015 Apr 16;11(4):e1005112. doi: 10.1371/journal.pgen.1005112 (PMC4400020; doi:10.1371/journal.pgen.1005112)
Supplement: S23 Table — UPI frequency (recomb.) is given by P(U 1 B 2) + P(U 2 B 1) + P(U 1 U 2)(1 – P b) (at equilibrium), while the UPI frequency (no mating types) is given by P(UB) + P(UU)(1 – P b) (at equilibrium). Additional parameters: P r = 0.5 (for recombination). See S5 Model for how we determined whether or not uniparental inheritance was maximized. (PDF) [file pgen.1005112.s037.pdf]

n	μ	Fitness	c_h	P_b	UPI frequency (recomb.)	UPI frequency (no mating types)	UPI maximized?
100	10^{-4}	concave	0.2	0.05	0.9524	0.9524	YES
100	10^{-4}	concave	0.2	0.25	0.6309	0.6309	NO
100	10^{-4}	concave	0.2	0.5	0.2044	0.2044	NO
100	10^{-4}	linear	0.2	0.05	0.9524	0.9524	YES
100	10^{-4}	linear	0.2	0.25	0.8000	0.8000	YES
100	10^{-4}	linear	0.2	0.5	0.6667	0.6667	YES
100	10^{-4}	convex	0.2	0.05	0.9524	0.9524	YES
100	10^{-4}	convex	0.2	0.25	0.8000	0.8000	YES
100	10^{-4}	convex	0.2	0.5	0.6667	0.6667	YES
