# Supplementary material for: Selection against Heteroplasmy Explains the Evolution of Uniparental Inheritance of Mitochondria
Source: PLoS Genet. 2015 Apr 16;11(4):e1005112. doi: 10.1371/journal.pgen.1005112 (PMC4400020; doi:10.1371/journal.pgen.1005112)
Supplement: S24 Table — Generations means the number of generations to reach equilibrium. UPI frequency is the frequency of uniparental inheritance at equilibrium. In rows 7, 8 and 10, in which there are few mitochondria, multiple mitotic divisions, and selection against heteroplasmy after mitosis, U 1 has no selective advantage and does not spread beyond its introductory frequency (when U 1 is introduced at a frequency of 0.01, the frequency of UPI is 0.02). Under these conditions, a mutation for uniparental inheritance could only spread via genetic drift; thus, biparental inheritance would be expected to remain stable if it were the ancestral condition. *The simulation in row 5 was stopped after 2 billion generations (before reaching equilibrium); while the spread of UPI was slowed in this simulation, it was not stopped. (PDF) [file pgen.1005112.s038.pdf]

n	μ	Fitness	c_h	Mitotic divisions	Generations	UPI frequency
4	10^{-7}	concave	0.01	0	173,046	1
4	10^{-7}	concave	0.5	0	53,972	1
4	10^{-7}	concave	0.01	20	3,880,317	1
4	10^{-7}	concave	0.5	20	483,751	1
4	10^{-7}	concave	0.01	50	$2x10^{9}$*	0.4036*
4	10^{-7}	concave	0.5	50	160,940,152	1
4	10^{-7}	concave	0.01	100	55,997,598	0.020000000295984
4	10^{-7}	concave	0.5	100	53,998,419	0.020000049257508
8	10^{-7}	concave	0.5	100	71,845,393	1
8	10^{-7}	concave	0.5	300	53,996,642	0.019999995259774
