# Supplementary material for: Selection against Heteroplasmy Explains the Evolution of Uniparental Inheritance of Mitochondria
Source: PLoS Genet. 2015 Apr 16;11(4):e1005112. doi: 10.1371/journal.pgen.1005112 (PMC4400020; doi:10.1371/journal.pgen.1005112)
Supplement: S25 Table — In this case, we apply selection after cells have gone through half of their mitotic divisions. After selection, we apply the second half of the mitotic divisions (e.g. in row one: 10 divisions, selection, 10 divisions). (PDF) [file pgen.1005112.s039.pdf]

n	μ	Fitness	c_h	Mitotic divisions	Generations	UPI frequency
4	10^{-7}	concave	0.01	20	815,514	1
4	10^{-7}	concave	0.5	20	122,471	1
4	10^{-7}	concave	0.5	100	160,940,164	1
