# Supplementary material for: Selection against Heteroplasmy Explains the Evolution of Uniparental Inheritance of Mitochondria
Source: PLoS Genet. 2015 Apr 16;11(4):e1005112. doi: 10.1371/journal.pgen.1005112 (PMC4400020; doi:10.1371/journal.pgen.1005112)
Supplement: S26 Table — Generations means the number of generations to reach equilibrium. UPI frequency is the frequency of uniparental inheritance at equilibrium. UPI frequency is given by P(U 1 B 2)(1 – P b) (at equilibrium). (PDF) [file pgen.1005112.s040.pdf]

n	μ	Fitness	c_h	P_b	Generations	U_1B_2 frequency	UPI frequency
20	10^{-4}	concave	0.2	0.01	21,884	1	0.99
20	10^{-4}	concave	0.2	0.05	523,616	0.0846	0.0804
20	10^{-4}	concave	0.2	0.25	25,815	zero	zero
20	10^{-4}	concave	0.2	0.5	23,666	zero	zero
20	10^{-4}	concave	0.2	0.75	45,258	zero	zero
20	10^{-4}	concave	0.2	0.99	960,209	zero	zero
20	10^{-4}	linear	0.2	0.01	19,923	1	0.99
20	10^{-4}	linear	0.2	0.05	78,331	1	0.95
20	10^{-4}	linear	0.2	0.25	297,201	1	0.75
20	10^{-4}	linear	0.2	0.5	615,848	1	0.5
20	10^{-4}	linear	0.2	0.75	1,314,439	1	0.25
20	10^{-4}	linear	0.2	0.99	26,893,152	1	0.01
20	10^{-4}	convex	0.2	0.01	20,312	1	0.99
20	10^{-4}	convex	0.2	0.05	51,748	1	0.95
20	10^{-4}	convex	0.2	0.25	84,089	1	0.75
20	10^{-4}	convex	0.2	0.5	109,360	1	0.5
20	10^{-4}	convex	0.2	0.75	178,889	1	0.25
20	10^{-4}	convex	0.2	0.99	3,219,783	1	0.01
