# Supplementary material for: Selection against Heteroplasmy Explains the Evolution of Uniparental Inheritance of Mitochondria
Source: PLoS Genet. 2015 Apr 16;11(4):e1005112. doi: 10.1371/journal.pgen.1005112 (PMC4400020; doi:10.1371/journal.pgen.1005112)
Supplement: S27 Table — We generated pseudo-random parameter values for P b, PU1 and PU2 using the 'twister' MATLAB rng. The rng values were normalized so that they sum to 1 because Pb+PU1+PU2=1. UPI(U 1) is given by PU1(U1U2)+U1B2, UPI(U 2) is given by PU2(U1U2)+U2B1 and BPI is given by P b(U 1 U 2)+B 1 B 2. (PDF) [file pgen.1005112.s041.pdf]

n	μ	Fitness	c_h	Random parameter values*	Inheritance pattern
20	10^{-4}	linear	0.2	$P_{U_1} = 0.1005$<br>$P_{U_2} = 0.8518$<br>$P_b = 0.0478$	$\text{UPI}(U_1): 0.1351$<br>$\text{UPI}(U_2): 0.8294$<br>$\text{BPI}: 0.0456$
20	10^{-4}	linear	0.2	$P_{U_1} = 0.5133$<br>$P_{U_2} = 0.4464$<br>$P_b = 0.0403$	$\text{UPI}(U_1): 0.5115$<br>$\text{UPI}(U_2): 0.4497$<br>$\text{BPI}: 0.0387$
20	10^{-4}	linear	0.2	$P_{U_1} = 0.4380$<br>$P_{U_2} = 0.4550$<br>$P_b = 0.1069$	$\text{UPI}(U_1): 0.4447$<br>$\text{UPI}(U_2): 0.4586$<br>$\text{BPI}: 0.0966$
20	10^{-4}	linear	0.2	$P_{U_1} = 0.3097$<br>$P_{U_2} = 0.2817$<br>$P_b = 0.4086$	$\text{UPI}(U_1): 0.3620$<br>$\text{UPI}(U_2): 0.3479$<br>$\text{BPI}: 0.2901$
20	10^{-4}	linear	0.2	$P_{U_1} = 0.4077$<br>$P_{U_2} = 0.4337$<br>$P_b = 0.1586$	$\text{UPI}(U_1): 0.4219$<br>$\text{UPI}(U_2): 0.4412$<br>$\text{BPI}: 0.1369$
20	10^{-4}	linear	0.2	$P_{U_1} = 0.4539$<br>$P_{U_2} = 0.4375$<br>$P_b = 0.1086$	$\text{UPI}(U_1): 0.4577$<br>$\text{UPI}(U_2): 0.4443$<br>$\text{BPI}: 0.0980$
20	10^{-4}	linear	0.2	$P_{U_1} = 0.0754$<br>$P_{U_2} = 0.3160$<br>$P_b = 0.6085$	$\text{UPI}(U_1): 0.2643$<br>$\text{UPI}(U_2): 0.3573$<br>$\text{BPI}: 0.3783$
20	10^{-4}	linear	0.2	$P_{U_1} = 0.2961$<br>$P_{U_2} = 0.5092$<br>$P_b = 0.1947$	$\text{UPI}(U_1): 0.23439$<br>$\text{UPI}(U_2): 0.4932$<br>$\text{BPI}: 0.1630$
20	10^{-4}	linear	0.2	$P_{U_1} = 0.4968$<br>$P_{U_2} = 0.1687$<br>$P_b = 0.3346$	$\text{UPI}(U_1): 0.4668$<br>$\text{UPI}(U_2): 0.2826$<br>$\text{BPI}: 0.2507$
20	10^{-4}	linear	0.2	$P_{U_1} = 0.2742$<br>$P_{U_2} = 0.3495$<br>$P_b = 0.3763$	$\text{UPI}(U_1): 0.3434$<br>$\text{UPI}(U_2): 0.3832$<br>$\text{BPI}: 0.2734$
