# Supplementary material for: Selection against Heteroplasmy Explains the Evolution of Uniparental Inheritance of Mitochondria
Source: PLoS Genet. 2015 Apr 16;11(4):e1005112. doi: 10.1371/journal.pgen.1005112 (PMC4400020; doi:10.1371/journal.pgen.1005112)
Supplement: S28 Table — (PDF) [file pgen.1005112.s042.pdf]

Variable	Description
a	the number (out of i) of type I mitochondria that mutate
b	the number (out of j) of type J mitochondria that mutate
c	the number (out of k) of type K mitochondria that mutate
x_i	the number (out of a) of mutations from type I mitochondria to type J mitochondria
x_j	the number (out of b) of mutations from type J mitochondria to type I mitochondria
x_k	the number (out of c) of mutations from type K mitochondria to type I mitochondria
$a - x_i$	the number (out of a) of mutations from type I mitochondria to type K mitochondria
$b - x_j$	the number (out of b) of mutations from type J mitochondria to type K mitochondria
$c - x_k$	the number (out of c) of mutations from type K mitochondria to type J mitochondria
