# Supplementary material for: Selection against Heteroplasmy Explains the Evolution of Uniparental Inheritance of Mitochondria
Source: PLoS Genet. 2015 Apr 16;11(4):e1005112. doi: 10.1371/journal.pgen.1005112 (PMC4400020; doi:10.1371/journal.pgen.1005112)
Supplement: S29 Table — Parameters: n = 20. (PDF) [file pgen.1005112.s043.pdf]

	t	v	y	z
Concave $(c_h = 0.1)$	0.25	0.68	2.5	0.9
Convex $(c_h = 0.1)$	0.05	1.5	1	0.9
