# Supplementary material for: Selection against Heteroplasmy Explains the Evolution of Uniparental Inheritance of Mitochondria
Source: PLoS Genet. 2015 Apr 16;11(4):e1005112. doi: 10.1371/journal.pgen.1005112 (PMC4400020; doi:10.1371/journal.pgen.1005112)
Supplement: S30 Table — (PDF) [file pgen.1005112.s044.pdf]

	ϕ	φ
$s_{d/a} = 10^{-2}$	0.2	0.2089
$s_{d/a} = 10^{-3}$	0.0628	0.063
$s_{d/a} = 10^{-4}$	0.0198	0.0199
$s_{d/a} = 10^{-5}$	0.00628	0.00628
