# Supplementary material for: Selection against Heteroplasmy Explains the Evolution of Uniparental Inheritance of Mitochondria
Source: PLoS Genet. 2015 Apr 16;11(4):e1005112. doi: 10.1371/journal.pgen.1005112 (PMC4400020; doi:10.1371/journal.pgen.1005112)
Supplement: S1 Text — Here we describe in more detail the simulation depicted in Figs. 2 and 3. (PDF) [file pgen.1005112.s045.pdf]

**S1 Text: Detailed model dynamics**

Once $B_1$ and $B_2$ gametes have reached mutation-selection equilibrium, part of the population is heteroplasmic (mutation-selection equilibrium is generation 0 in Figs. 2 and 3). When a mutation from $B_1$ to $U_1$ occurs in a gamete homoplasmic for the wild type haplotype, the proportion of $B_1$ and $B_2$ gametes with any level of heteroplasmy initially decreases (generations 0 – 100 in Figs. 2C and 3C-D). The influx of $U_1$ gametes homoplasmic for the wild type haplotype converts some heteroplasmic $B_2$ gametes into homoplasmic $B_2$ gametes. In turn, this drives down the proportion of heteroplasmy in $B_1$ gametes via $B_1 \times B_2$ matings. (When the mutation rate is smaller, this initial drop in heteroplasmy is less noticeable (S2 and S3 Figs.).)

After about 100 generations, $U_1$ gametes homoplasmic for mutant mitochondria begin to increase in frequency (Fig. 3B). As described earlier, this leads to matings between $B_2$ gametes homoplasmic for mutant mitochondria and $B_1$ gametes carrying the wild type haplotype, which result in heteroplasmic $B_1B_2$ cells (Figs. 2C and 3C-D; note that most $B_1$ gametes are homoplasmic for the wild type haplotype or only carry a few mutant mitochondria at this stage). This results in an increase in the proportion of heteroplasmic $B_1$ and $B_2$ gametes and $B_1B_2$ cells (generations 100 – 1350 in Figs. 2C and 3C-D). Selection against heteroplasmy thus decreases the relative fitness of $B_1$ ( $\overline{w}_{B_1}$) and $B_2$ ($\overline{w}_{B_2}$) gametes and $B_1B_2$ ($\overline{w}_{B_1B_2}$) cells (Figs. 2A and 3A). From generations 1350 – 1820, the proportion of heteroplasmic $B_1$ and $B_2$ gametes and $B_1B_2$ cells decreases (Figs. 2C and 3C-D). Despite this, $\overline{w}_{B_1}$ and $\overline{w}_{B_1B_2}$ continue to decrease ($\overline{w}_{B_2}$, however, starts to converge with $\overline{w}_{U_1}$). While the proportion of heteroplasmic $B_1$ and $B_2$ gametes and $B_1B_2$ cells decreases during this period, the

level of heteroplasmy within heteroplasmic gametes and cells increases (Figs. 2C-E and 3C-F). The increased levels of heteroplasmy outweigh the reduced proportion of heteroplasmic cells, and the net effect is increased selection against heteroplasmic $B_1B_2$ cells (Figs. 2A, 3A).

From generations 1350 – 1820 $U_1$ rapidly spreads through the population, increasing from 0.077 to 0.474. During this period, $U_1 \times B_2$ matings become more frequent, increasing the proportion of homoplasmic $B_2$ gametes. In turn, this increases the proportion of homoplasmic $B_1B_2$ cells and $B_1$ gametes through $B_1 \times B_2$ matings (Figs. 2C-E and 3C-F). More $B_2$ gametes are now homoplasmic for mutant mitochondria (Fig. 3D; note that these $B_2$ gametes begin to appear around generation 1400 in Fig. 3D). $B_1 \times B_2$ matings involving $B_2$ gametes homoplasmic for mutant mitochondria become more common, leading to $B_1B_2$ cells with high levels of heteroplasmy (compare Fig. 2D with 2E). Increased levels of heteroplasmy within $B_1B_2$ cells drives down $\overline{w}_{B_1B_2}$ and $\overline{w}_{B_1}$ (Figs. 2A and 3A). As the frequency of $B_1$ decreases, $\overline{w}_{B_2}$ becomes increasingly determined by $U_1 \times B_2$ matings and $\overline{w}_{B_2}$ converges to $\overline{w}_{U_1}$ around generation 1900 (Fig. 3A). During the remainder of the simulation, $\overline{w}_{B_1B_2}$ and $\overline{w}_{B_1}$ decrease further as $U_1$ replaces $B_1$.

Since there are few cells homoplasmic for mutant mitochondria at the beginning of the simulation, the relative advantage of $U_1$ over $B_1$ is low when the frequency of $U_1$ is low (e.g. $\overline{w}_{U_1} = 0.99984$ and $\overline{w}_{B_1} = 0.99881$ at generation 50 in Fig. 3A, giving a relative advantage for $U_1$ of 0.001). As the frequency of $U_1$ gametes with mutant mitochondria increases, so too does the relative advantage of $U_1$ (e.g. $\overline{w}_{U_1} = 0.99984$

and $\overline{w}_{B_1} = 0.98476$ at generation 2500 in Fig. 3A, giving a relative advantage for $U_1$ of 0.015). For more details about the change in gamete and cell type distributions as $U_1$ spreads, see S1-S2 Videos.
