# Supplementary material for: Selection against Heteroplasmy Explains the Evolution of Uniparental Inheritance of Mitochondria
Source: PLoS Genet. 2015 Apr 16;11(4):e1005112. doi: 10.1371/journal.pgen.1005112 (PMC4400020; doi:10.1371/journal.pgen.1005112)
Supplement: S2 Text — Additional detail for the no mating types scenario. (PDF) [file pgen.1005112.s046.pdf]

S2 Text: No mating types

In the absence of mating types, there are two gametes (B and U) and three genotypes (BB, UB and UU). As in the recombination case, when $U \times U$ matings lead to uniparental inheritance, the UU genotype always spreads until it is fixed in the population, leading to complete uniparental inheritance (S16-S18 Tables). When $U \times U$ matings lead to biparental inheritance or a mixture of uniparental inheritance and biparental inheritance, uniparental inheritance does not become fixed (again, as in the recombination case) (S19-S23 Tables). The only difference between the no mating type and recombination scenarios is that the UB genotype (no mating types) has the same frequency as the sum of the U_1B_2 and U_2B_1 genotypes (recombination) at equilibrium (provided that P_r is sufficiently large) (Fig. 4A,F and S16-S23 Tables). Thus, the no mating type case can be inferred from the recombination case in the main text by setting $P(UB) = P(U_1B_2) + P(U_2B_1)$.
