# Supplementary material for: Selection against Heteroplasmy Explains the Evolution of Uniparental Inheritance of Mitochondria
Source: PLoS Genet. 2015 Apr 16;11(4):e1005112. doi: 10.1371/journal.pgen.1005112 (PMC4400020; doi:10.1371/journal.pgen.1005112)
Supplement: S1 Model — (PDF) [file pgen.1005112.s047.pdf]

S1 Model: Non-neutral models

Deleterious model

Let s_d be the selection coefficient of the deleterious mutation (where the fitness of a cell that is homoplasmic for mutant mitochondria is $1-s_d$). Assuming that the cost imposed by mutant mitochondria can be modeled by a concave function, the fitness of a cell with i mutant mitochondria is given by

$w_d(i)= w(i)\left(1-s_d\left(\frac{i}{n}\right)^2\right)$, where $w(i)$ is determined by either equation (1) or (3).

Advantageous model

Again, we generate $w(i)$ using equation (1) or equation (3). We let s_a be the selection coefficient of the deleterious mutation (where the fitness of a cell that is homoplasmic for wild type mitochondria is $1-s_a$). Since there are no data as to how fitness increases as advantageous mitochondria accumulate, we model the increase in fitness using a concave function and a convex function. If we assume that advantageous mutations convey fitness benefits consistent with a concave function, the fitness of a cell with i mutant mitochondria is given by

$$w_a(i)= w(i)\left(1-s_a\left(\frac{n-i}{n}\right)^2\right).$$

If we assume that fitness accumulates as a convex function of the number of mutant mitochondria, then the fitness of a cell with i mutant mitochondria is given by

$$w_a(i) = w(i)\left(1-s_d\sqrt{\frac{n-i}{n}}\right).$$
