# Supplementary material for: Selection against Heteroplasmy Explains the Evolution of Uniparental Inheritance of Mitochondria
Source: PLoS Genet. 2015 Apr 16;11(4):e1005112. doi: 10.1371/journal.pgen.1005112 (PMC4400020; doi:10.1371/journal.pgen.1005112)
Supplement: S2 Model — (PDF) [file pgen.1005112.s048.pdf]

S2 Model: Mating types with recombination

In this scenario, there are four gametes (U_1, U_2, B_1 and B_2) and four genotypes (U_1U_2, U_1B_2, U_2B_1 and B_1B_2).

Initialization

We introduce the U_1 allele into homoplasmic wild type gametes at a proportion of 0.01 and remove 0.01 from the B gametes. All other details of initialization remain the same as the general model.

Random mating

Biparental mating (B_1B_2 ***cells)***

The probability of producing a B_1B_2 cell type after random mating is given by

$$P\left(\mathbf{M}^{t,\tau_2}=\left(i,B_1B_2\right)\right)=\sum_{p=\max(0,i-n/2)}^{\min(n/2,i)} P\left(\mathbf{M}^{t,\tau_1}=\left(p,B_1\right)\right)P\left(\mathbf{M}^{t,\tau_1}=\left(i-p,B_2\right)\right).$$

Biparental mating (U_1U_2 ***cells)***

The probability of producing a U_1U_2 cell, when we assume that U_1U_2 matings are biparental, is given by

$$P\left(\mathbf{M}^{t,\tau_2}=\left(i,U_1U_2\right)\right)=\sum_{p=\max(0,i-n/2)}^{\min(n/2,i)} P\left(\mathbf{M}^{t,\tau_1}=\left(p,U_1\right)\right)P\left(\mathbf{M}^{t,\tau_1}=\left(i-p,U_2\right)\right).$$

Uniparental mating (U_1B_2 ***and*** U_2B_1 ***cells)***

The probability of forming a U_1B_2 cell is

$$P\left(\mathbf{M}^{t,\tau_2}=\left(i,U_1B_2\right)\right)=\sum_{p=\max\left(0,i-\frac{n}{2}\right)}^{\min\left(\frac{n}{2},i\right)} P\left(\mathbf{M}^{t,\tau_1}=\left(p,U_1\right)\right)\,T\left(i-p;\frac{n}{2},\frac{2p}{n}\right)\sum_{r=0}^{\frac{n}{2}}P\left(\mathbf{M}^{t,\tau_1}=\left(r,B_2\right)\right),$$

and the probability of producing a U_2B_1 cell is

$$P\left(\mathbf{M}^{t,\tau_2}=(i,U_2B_1)\right)=\sum_{p=\max\left(0,i-\frac{n}{2}\right)}^{\min\left(\frac{n}{2},i\right)} P\left(\mathbf{M}^{t,\tau_1}=(p,U_2)\right)\ T\left(i-p;\frac{n}{2},\frac{2p}{n}\right)\sum_{r=0}^{\frac{n}{2}} P\left(\mathbf{M}^{t,\tau_1}=(r,B_1)\right).$$

Uniparental mating (U_1U_2 cells)

The probability of producing a U_1U_2 cell, when we assume that U_1U_2 matings are uniparental, is

$$P\left(\mathbf{M}^{t,\tau_2}=(i,U_1U_2)\right)=\sum_{p=\max\left(0,i-\frac{n}{2}\right)}^{\min\left(\frac{n}{2},i\right)} P\left(\mathbf{M}^{t,\tau_1}=(p,U_1)\right)\ T\left(i-p;\frac{n}{2},\frac{2p}{n}\right)\sum_{r=0}^{\frac{n}{2}} P\left(\mathbf{M}^{t,\tau_1}=(r,U_2)\right).$$

(Note that we also ran simulations where U_2, rather than U_1, was the mitochondrial donor but this did not affect our results.) As before, mutation, selection and normalization are the same as the general model.

Meiosis

During meiosis 1, homologous chromosomes line up and may undergo recombination. The probability of recombination, P_r, cannot exceed 0.5 because, at most, only two of the four chromatids can recombine. U_2 gametes are produced when U_1B_2 cells undergo recombination, giving rise to a U_2B_1 cell. U_2B_1 cells may also undergo recombination to give U_1B_2 cells. If we let P_r be the probability that the mating type and inheritance loci recombine, then the probability of producing a U_1 gamete is given by

$$P\left(\mathbf{M}^{t+1,\tau_1}=(p,U_1)\right)=\frac{1}{2}(1-P_r)\left(\sum_{l=0}^{2n} S\left(p;2n,l,\frac{n}{2}\right)P\left(\mathbf{M}^{t,\tau_6}=(l,U_1U_1B_2B_2)\right)\right)$$
$$+\frac{1}{2}P_r\left(\sum_{l=0}^{2n} S\left(p;2n,l,\frac{n}{2}\right)P\left(\mathbf{M}^{t,\tau_6}=(l,U_2U_2B_1B_1)\right)\right)+\frac{1}{2}\left(\sum_{l=0}^{2n} S\left(p;2n,l,\frac{n}{2}\right)P\left(\mathbf{M}^{t,\tau_6}=(l,U_1U_1U_2U_2)\right)\right),$$

the probability of producing a U_2 gamete is

$$P\left(\mathbf{M}^{t+1,\tau_1}=\left(p,U_2\right)\right)=\frac{1}{2}\left(1-P_r\right)\left(\sum_{l=0}^{2n}S\left(p;2n,l,\frac{n}{2}\right)P\left(\mathbf{M}^{t,\tau_6}=\left(l,U_2U_2B_1B_1\right)\right)\right)$$
$$+\frac{1}{2}P_r\left(\sum_{l=0}^{2n}S\left(p;2n,l,\frac{n}{2}\right)P\left(\mathbf{M}^{t,\tau_6}=\left(l,U_1U_1B_2B_2\right)\right)\right)+\frac{1}{2}\left(\sum_{l=0}^{2n}S\left(p;2n,l,\frac{n}{2}\right)P\left(\mathbf{M}^{t,\tau_6}=\left(l,U_1U_1U_2U_2\right)\right)\right),$$

the probability of producing a B_1 gamete is

$$P\left(\mathbf{M}^{t+1,\tau_1}=\left(p,B_1\right)\right)=\frac{1}{2}\left(1-P_r\right)\left(\sum_{l=0}^{2n}S\left(p;2n,l,\frac{n}{2}\right)P\left(\mathbf{M}^{t,\tau_6}=\left(l,U_2U_2B_1B_1\right)\right)\right)$$
$$+\frac{1}{2}P_r\left(\sum_{l=0}^{2n}S\left(p;2n,l,\frac{n}{2}\right)P\left(\mathbf{M}^{t,\tau_6}=\left(l,U_1U_1B_2B_2\right)\right)\right)+\frac{1}{2}P_r\left(\sum_{l=0}^{2n}S\left(p;2n,l,\frac{n}{2}\right)P\left(\mathbf{M}^{t,\tau_6}=\left(l,B_1B_1B_2B_2\right)\right)\right),$$

and the probability of producing a B_2 gamete is

$$P\left(\mathbf{M}^{t+1,\tau_1}=\left(p,B_2\right)\right)=\frac{1}{2}\left(1-P_r\right)\left(\sum_{l=0}^{2n}S\left(p;2n,l,\frac{n}{2}\right)P\left(\mathbf{M}^{t,\tau_6}=\left(l,U_1U_1B_2B_2\right)\right)\right)$$
$$+\frac{1}{2}P_r\left(\sum_{l=0}^{2n}S\left(p;2n,l,\frac{n}{2}\right)P\left(\mathbf{M}^{t,\tau_6}=\left(l,U_2U_2B_1B_1\right)\right)\right)+\frac{1}{2}\left(\sum_{l=0}^{2n}S\left(p;2n,l,\frac{n}{2}\right)P\left(\mathbf{M}^{t,\tau_6}=\left(l,B_1B_1B_2B_2\right)\right)\right).$$
