# Supplementary material for: Selection against Heteroplasmy Explains the Evolution of Uniparental Inheritance of Mitochondria
Source: PLoS Genet. 2015 Apr 16;11(4):e1005112. doi: 10.1371/journal.pgen.1005112 (PMC4400020; doi:10.1371/journal.pgen.1005112)
Supplement: S3 Model — (PDF) [file pgen.1005112.s049.pdf]

S3 Model: No mating types scenario

In this version of the model, there are two possible alleles (U and B) and three possible genotypes (UU, UB and BB).

Initialization

We introduce the U allele into homoplasmic wild type gametes at a proportion of 0.01 and remove 0.01 from the B gametes. All other details of initialization remain the same as the general model.

Random mating

Biparental mating (BB cells)

The probability of producing a BB cell type after random mating is given by

$$P\left(\mathbf{M}^{t,\tau_2} = (i,BB)\right) = \sum_{p=\max(0,i-n/2)}^{\min(n/2,i)} P\left(\mathbf{M}^{t,\tau_1} = (p,B)\right)P\left(\mathbf{M}^{t,\tau_1} = (i-p,B)\right).$$

Biparental mating (UU cells)

The probability of producing a UU cell, when we assume that UU matings are biparental, is given by

$$P\left(\mathbf{M}^{t,\tau_2} = (i,UU)\right) = \sum_{p=\max(0,i-n/2)}^{\min(n/2,i)} P\left(\mathbf{M}^{t,\tau_1} = (p,U)\right)P\left(\mathbf{M}^{t,\tau_1} = (i-p,U)\right).$$

Uniparental mating (UB cells)

The probability of forming a UB cell by random mating is determined by

$$P\left(\mathbf{M}^{t,\tau_2} = (i,UB)\right) = 2\left(\sum_{p=\max\left(0,i-\frac{n}{2}\right)}^{\min\left(\frac{n}{2},i\right)} P\left(\mathbf{M}^{t,\tau_1} = (p,U)\right)T\left(i-p;\frac{n}{2},\frac{2p}{n}\right)\sum_{r=0}^{\frac{n}{2}} P\left(\mathbf{M}^{t,\tau_1} = (r,B)\right)\right).$$

Uniparental mating (UU cells)

The probability of producing a UU cell, when we assume that UU matings are uniparental, is

$$P\left(\mathbf{M}^{t,\tau_2}=(i,UU)\right)=\sum_{p=\max\left(0,i-\frac{n}{2}\right)}^{\min\left(\frac{n}{2},i\right)} P\left(\mathbf{M}^{t,\tau_1}=(p,U)\right)\, T\left(i-p;\frac{n}{2},\frac{2p}{n}\right)\sum_{r=0}^{\frac{n}{2}} P\left(\mathbf{M}^{t,\tau_1}=(r,U)\right).$$

The mutation, selection and normalization stages are the same as the general model (although there are now three genotypes instead of two).

Meiosis

The process by which cells in state $\mathbf{M}^{t,\tau_5}=(i,G)$ become cells in state $\mathbf{M}^{t,\tau_6}=(l,2G)$ does not change. Thus, the probability of producing a U gamete is

$$P\left(\mathbf{M}^{t+1,\tau_1}=(p,U)\right)=\frac{1}{2}\left(\sum_{l=0}^{2n} S\left(p;2n,l,\frac{n}{2}\right)P\left(\mathbf{M}^{t,\tau_6}=(l,UUBB)\right)\right)+\left(\sum_{l=0}^{2n} S\left(p;2n,l,\frac{n}{2}\right)P\left(\mathbf{M}^{t,\tau_6}=(l,UUUU)\right)\right),$$

while the probability of producing a B gamete is

$$P\left(\mathbf{M}^{t+1,\tau_1}=(p,B)\right)=\frac{1}{2}\left(\sum_{l=0}^{2n} S\left(p;2n,l,\frac{n}{2}\right)P\left(\mathbf{M}^{t,\tau_6}=(l,UUBB)\right)\right)+\left(\sum_{l=0}^{2n} S\left(p;2n,l,\frac{n}{2}\right)P\left(\mathbf{M}^{t,\tau_6}=(l,BBBB)\right)\right).$$
