# Supplementary material for: Selection against Heteroplasmy Explains the Evolution of Uniparental Inheritance of Mitochondria
Source: PLoS Genet. 2015 Apr 16;11(4):e1005112. doi: 10.1371/journal.pgen.1005112 (PMC4400020; doi:10.1371/journal.pgen.1005112)
Supplement: S4 Model — (PDF) [file pgen.1005112.s050.pdf]

S4 Model: Mitosis

In this scenario, we add mitosis to the life-cycle. Mitosis mimics the continual turnover of mitochondria that occurs within a cell. (Mitochondria form interconnected networks that continually undergo fission/fusion.) We examine how the model behaves when mitosis is inserted before selection and when it is inserted after selection. To model mitosis, we sample n mitochondria with replacement from a cell that contains i mutant (out of n) mitochondria. We denote the probability of sampling $i`$ mutant mitochondria with replacement from a cell with $i$ mutant mitochondria as $X\left(i`;n,i/n\right)$. When mitosis occurs before selection, the post-mitosis population is determined by

$$P\left(\mathbf{M}^{t,\tau_4}=\left(i`,U_1B_2\right)\right)=\sum_{i=0}^{n}X\left(i`;n,\frac{i}{n}\right)P\left(\mathbf{M}^{t,\tau_3}=\left(i,U_1B_2\right)\right).$$

When mitosis occurs after selection, the post-mitosis population is given by

$$P\left(\mathbf{M}^{t,\tau_6}=\left(i`,U_1B_2\right)\right)=\sum_{i=0}^{n}X\left(i`;n,\frac{i}{n}\right)P\left(\mathbf{M}^{t,\tau_5}=\left(i,U_1B_2\right)\right).$$
