# Supplementary material for: Selection against Heteroplasmy Explains the Evolution of Uniparental Inheritance of Mitochondria
Source: PLoS Genet. 2015 Apr 16;11(4):e1005112. doi: 10.1371/journal.pgen.1005112 (PMC4400020; doi:10.1371/journal.pgen.1005112)
Supplement: S5 Model — (PDF) [file pgen.1005112.s051.pdf]

S5 Model: Frequencies of U and B alleles that maximize the level of uniparental inheritance

Here we determine the frequencies of U and B that maximize the level of uniparental inheritance, assuming that $U \times U$ matings have biparental inheritance with probability P_b $(0 \leq P_b \leq 1)$ and uniparental inheritance with probability $1 - P_b$. Writing U as the total proportion of the population with the U allele and B as the total proportion of the population with the B allele, the pre-mating population of gametes satisfies:

$U + B = 1$,

and the post-mating population satisfies:

$$P_b U^2 + (1 - P_b)U^2 + 2UB + B^2 = 1. \quad (4)$$

We define the part of equation (4) that leads to uniparental inheritance as $f = (1 - P_b)U^2 + 2UB$. Thus we can rearrange equation (4) to give

$$f = 1 - P_b U^2 - B^2. \quad (5)$$

We substitute $B = 1 - U$ into equation (5) to give

$$f(B) = 1 - P_b(1 - B)^2 - B^2,$$

which upon rearrangement gives

$$f(B) = (1 - P_b) + 2P_b B - (P_b + 1)B^2. \quad (6)$$

Differentiating equation (6) with respect to B gives:

$$\frac{df}{dB} = 2P_b - 2(P_b + 1)B. \quad (7)$$

Local optima of $f(B)$ satisfy $\frac{df}{dB} = 0$. Therefore,

$$2P_b = 2(P_b + 1)B,$$

which leads to

$$B = \frac{P_b}{P_b + 1}. \qquad (8)$$

The frequency of the U allele at equilibrium is then $U = 1 - B$.

Differentiating equation (7) with respect to B gives

$$\frac{d^2 f}{dB^2} = -2(P_b + 1) ,$$

which is less than 0 for all $P_b \in [0,1]$. Hence the optima at $B = P_b / (P_b + 1)$ is a local maximum. At the local maximum,

$$f(B_{\max}) = \frac{1}{P_b + 1}.$$

To determine if the local maximum also indicates the maximum value of $f(B)$ we must also check the values of $f(B)$ at the end points of the line $U + B = 1$. $f(1) = 0$ and $f(0) = 1 - P_b$. For all $P_b \in [0,1]$, $f(0) \le f(B_{\max})$, and therefore the maximum frequency of uniparental inheritance occurs when $B = P_b / (P_b + 1)$.

We checked the predictions of equation (8) against our simulation results when $U \times U$ matings are possible. When fitness is linear or convex, equation (8) predicts the equilibrium state every time (*i.e.* the equilibrium state is such that the frequency of uniparental inheritance is at its maximum possible value). When fitness is concave, however, uniparental inheritance is only maximized under certain values of P_b (equation (8)) (S22-S23 Tables).
