# Supplementary material for: Selection against Heteroplasmy Explains the Evolution of Uniparental Inheritance of Mitochondria
Source: PLoS Genet. 2015 Apr 16;11(4):e1005112. doi: 10.1371/journal.pgen.1005112 (PMC4400020; doi:10.1371/journal.pgen.1005112)
Supplement: S6 Model — (PDF) [file pgen.1005112.s052.pdf]

S6 Model: Additional model that explores three mitochondrial types

Diploid cell types are described by the vector $\mathbf{M}^{t,\tau_\alpha} = (i, j, G)$, where i corresponds to the number of type I mitochondria and takes values in $\{0,1...n\}$, j represents the number of type J mitochondria and takes values in $\{0,1..n-i\}$, t indicates the generation, and τ_α indicates the stage of the life cycle. If i and j are specified, the number of type K mitochondria is fixed as $k = n - i - j$. G indicates the nuclear genotype and takes values in $\{U_1B_2, B_1B_2\}$. Gametes are described by the vector $\mathbf{M}^{t,\tau_\alpha} = (p, q, g)$, where p is the number of type I mitochondria and takes values in $\{0,1...n/2\}$, and q is the number of type J mitochondria and takes values in $\{0,1...n/2-p\}$. g represents the nuclear allele and takes values in $\{U_1, B_1, B_2\}$. The probability of obtaining a particular diploid cell type is written as $P\left(\mathbf{M}^{t,\tau_\alpha} = (i, j, G)\right)$ and the probability of obtaining a particular gamete is written as $P\left(\mathbf{M}^{t,\tau_\alpha} = (p, q, g)\right)$.

There are $\left((n+1)(n+2)\right)/2$ total mitochondrial states for diploid cells and $\left(((n/2)+1)((n/2)+2)\right)/2$ possible mitochondrial states for haploid cells.

Initialization

The starting population is evenly split between B_1 and B_2 gametes, and all gametes contain type K mitochondria (i.e. $P\left(\mathbf{M}^{0,\tau_1} = (0, 0, B_1)\right) = 0.5$, $P\left(\mathbf{M}^{0,\tau_1} = (0, 0, B_2)\right) = 0.5$ and $P\left(\mathbf{M}^{0,\tau_1} = (p, q, g)\right) = 0, \forall\, p > 0$, $q > 0$ and $g = U_1$). After 100 generations we identify the mitochondrial state of the B_1 gametes that makes up the greatest proportion of the population

(denoted $\mathbf{M}^{101,\tau_1} = \left(p^*, q^*, B_1\right)$). We reduce the proportion of this cell type by 0.01 $\left(\text{i.e. } P\left(\mathbf{M}^{101,\tau_1} = \left(p^*, q^*, B_1\right)\right) = P\left(\mathbf{M}^{101,\tau_1} = \left(p^*, q^*, B_1\right)\right) - 0.01\right)$ and then we introduce U_1 gametes by setting $P\left(\mathbf{M}^{101,\tau_1} = \left(p^*, q^*, U_1\right)\right) = 0.01$. Unless μ is very high, these conditions lead to U_1 being introduced where $p^* = q^* = 0$ (i.e. homoplasmic for type K).

Random mating

Biparental mating

Consider a biparental mating involving a gamete in state $\mathbf{M}^{t,\tau_1} = \left(p,q,B_1\right)$, where τ_1 is the gamete stage of the life cycle. For this gamete to produce a diploid cell with type $\mathbf{M}^{t,\tau_2} = \left(i,j,B_1B_2\right)$, where τ_2 is the diploid cell stage of the life cycle that precedes mutation, it must mate with a gamete of type $\mathbf{M}^{t,\tau_1} = \left(i-p, j-q, B_2\right)$. The probability of this mating is $2P\left(\mathbf{M}^{t,\tau_1} = \left(p,q,B_1\right)\right)P\left(\mathbf{M}^{t,\tau_1} = \left(i-p, j-q, B_2\right)\right)$. However, not all combinations of p, q, $i-p$ and $j-q$ lead to valid matings; thus, we must restrict these values to biologically valid combinations.

First, $0 \le p \le n/2$, as the B_1 gamete cannot carry negative numbers of type I mitochondria nor can it contain more type I mitochondria than the total number of mitochondria in the gamete. Likewise, $0 \le i-p \le n/2$ for the B_2 gamete, which rearranged gives $i-\left(n/2\right) \le p \le i$. Valid values for p lie in the range of intersection of these two inequalities, giving $\max\left(0, i-\left(n/2\right)\right) \le p \le \min\left(n/2, i\right)$.

The first restriction for q is $0 \le q \le \left(n/2\right) - p$ because the B_1 gamete already contains p type I mitochondria and cannot contain more than $n/2$ mitochondria. q is also restricted by $0 \le j-q \le \left(n/2\right) - \left(i-p\right)$ because the B_2 gamete cannot contain more than $n/2$

mitochondria and already contains $i-p$ type I mitochondria, which gives $i+j-(n/2)-p \le q \le j$ when rearranged. The intersection of these inequalities gives,

$$\max\left(0,i+j-(n/2)-p\right) \le q \le \min\left((n/2)-p,j\right).$$

Thus,

$$P\left(\mathbf{M}^{t,\tau_2}=(i,j,B_1B_2)\right)=2\left(\sum_{p=\max\left(0,i-\frac{n}{2}\right)}^{\min\left(\frac{n}{2},i\right)}\sum_{q=\max\left(0,i+j-\frac{n}{2}-p\right)}^{\min\left(\frac{n}{2}-p,j\right)} P\left(\mathbf{M}^{t,\tau_1}=(p,q,B_1)\right)P\left(\mathbf{M}^{t,\tau_1}=(i-p,j-q,B_2)\right)\right).$$

Uniparental mating

Since uniparental matings between U_1 and B_2 gametes contain mitochondria from U_1 alone, U_1B_2 cells initially have $n/2$ mitochondria. To restore the total complement of n mitochondria, we sample $n/2$ mitochondria with replacement from the $n/2$ mitochondria in the U_1B_2 cell and add the $n/2$ sampled mitochondria to the original set of mitochondria to form a cell with n mitochondria.

For a gamete with identity $\mathbf{M}^{t,\tau_1}=(p,q,U_1)$ to produce a diploid cell with identity $\mathbf{M}^{t,\tau_2}=(i,j,U_1B_2)$, it must sample $n/2$ mitochondria containing $i-p$ type I mitochondria and $j-q$ type J mitochondria. The mitochondrial state of the B_2 gamete is irrelevant because its mitochondria are discarded and we will refer to this cell as $\mathbf{M}^{t,\tau_1}=(r,s,B_2)$.

Sampling of the $n/2$ mitochondria follows a multinomial distribution, which we denote $T\left(i-p,j-q;n/2,(2p)/n,(2q)/n\right)$, where $i-p$ and $j-q$ refer to the number of type I and

J mitochondria that need to be sampled, $n/2$ refers to the number of mitochondria being sampled, and $(2p)/n$ and $(2q)/n$ refer to the probability of drawing type I and J mitochondria respectively from a U_1B_2 cell that contains p type I and q type J mitochondria and a total of $n/2$ mitochondria (where $(2p)/n$ is obtained by rearranging $p/(n/2)$ and $(2q)/n$ is obtained by rearranging $q/(n/2)$).

The probability of sampling $i-p$ and $j-q$ type I and J mitochondria respectively (and $(n/2)-(i-p)-(j-q)$ type K mitochondria) is given by

$$T\left(i-p,j-q;\frac{n}{2},\frac{2p}{n},\frac{2q}{n}\right)=\frac{\frac{n}{2}!}{(i-p)!(j-q)!\left(\frac{n}{2}-(i-p)-(j-q)\right)!}\left(\frac{2p}{n}\right)^{i-p}\left(\frac{2q}{n}\right)^{j-q}\left(\frac{2\left(\frac{n}{2}-p-q\right)}{n}\right)^{\frac{n}{2}-(i-p)-(j-q)}.$$

The restrictions on p, q, $i-p$ and $j-q$ are the same as those in biparental mating. Because U_1 will form the same initial U_1B_2 cell regardless of the B_2 gamete with which it mates, the probability of each U_1 gamete is multiplied by the probability of selecting each B_2 gamete. The probability of forming a given U_1B_2 cell after random mating is

$$P\left(\mathbf{M}^{t,\tau_2}=(i,j,U_1B_2)\right)=\sum_{p=\max\left(0,i-\frac{n}{2}\right)}^{\min\left(\frac{n}{2},i\right)}\sum_{q=\max\left(0,i+j-\frac{n}{2}-p\right)}^{\min\left(\frac{n}{2}-p,j\right)}\left(2P\left(\mathbf{M}^{t,\tau_1}=(p,q,U_1)\right)T\left(i-p,j-q;\frac{n}{2},\frac{2p}{n},\frac{2q}{n}\right)\sum_{r=0}^{\frac{n}{2}}\sum_{s=0}^{\frac{n}{2}-r}P\left(\mathbf{M}^{t,\tau_1}=(r,s,B_2)\right)\right).$$

Mutation

As each mitochondrion can mutate into either of the other two mitochondrial types, there can be many different transitions between pre-mutation cell types and a single specified post-mutation cell type, which we denote as $\mathbf{M}^{t,\tau_3}=(i,j,G)$, where τ_3 refers to the post-mutation

life cycle stage. We introduce six mutation variables $\left(a, b, c, x_i, x_j \text{ and } x_j\right)$ to describe the way in which a pre-mutation cell can mutate to a post-mutation cell (S28 Table).

Cells lose a type I mitochondria due to mutation from type I into other mitochondrial types, but concurrently gain $x_j + x_k$ type I mitochondria that have mutated away from type J and K. Thus, post-mutation cells with i type I mitochondria come from pre-mutation cells with $i + a - x_j - x_k$ type I mitochondria. Similarly, post-mutation cells with j type J mitochondria are derived from pre-mutation cells with $j + b - x_i - \left(c - x_k\right)$ type J mitochondria, and (implicitly) post-mutation cells with $k = n - i - j$ type K mitochondria are derived from pre-mutation cells with $k + c - (a - x_i) - (b - x_j)$ type K mitochondria. Thus, post-mutation cells in state $\mathbf{M}^{t,\tau_3} = \left(i, j, G\right)$ are derived from pre-mutation cells in state

$$\begin{aligned}\mathbf{M}^{t,\tau_2} &= \left(i + a - x_j - x_k, j + b - x_i - \left(c - x_k\right), G\right) \\ &= \left(i', j', G\right)\end{aligned}.$$

We identify all valid combinations of mutation variables that lead from pre-mutation cells to a particular post-mutation cell in state $\mathbf{M}^{t,\tau_3} = \left(i, j, G\right)$.

To determine allowable ranges for a, b, and c we use the following conceptual approach: the number of mutations away from a particular type must be less than or equal to the total number of mitochondria that can be received by the other two mitochondrial types.

Thus, a, the number of type I mitochondria that mutate into type J or type K mitochondria, must be less than or equal to the sum of j type J mitochondria and k type K mitochondria. Therefore, $a \le j + k$, where $j + k = j + (n - i - j) = n - i$, giving $0 \le a \le n - i$.

Before we can determine b (mutations away from type J), however, we must know how many of the a mutations in the type I mitochondria become type J (x_i), as x_i affects the restrictions on b.

Our conceptual approach for x_i, x_j and x_k is as follows. Each of these variables has three restrictions. First, by definition, these variables are less than or equal to the number of mutations in a particular mitochondrial type (e.g. x_i must satisfy $0 \le x_i \le a$). The second and third restrictions ensure that the number of mutations to a particular mitochondrial type is less than or equal to the number of mitochondria required by that mitochondrial type. Valid values of these variables are found within the range of intersection of these three restrictions.

The first restriction for x_i, the number (out of a) of type I mitochondria that mutate to type J, is $0 \le x_i \le a$. The second restriction is $x_i \le j$ (number of mutations to type J is less than or equal to the number of mutations required by type J) and the third restriction is $a - x_i \le k$ (number of mutations to type K is less than or equal to the number of mutations required by type K), which gives $x_i \ge a - n + i + j$. The intersection of these restrictions gives $\max(0, a - n + i + j) \le x_i \le \min(j, a)$.

Now we move to b, the number of type J mitochondria that mutate into type I or type K mitochondria. The mutations in type J cannot be greater than the number of type I and type

K mitochondria required by the cell post-mutation. Thus, $b \le i + k - (a - x_i)$, where i is the number of required type I mitochondria and $k - (a - x_i)$ is the number of type K mitochondria still required (because the cell receives $a - x_i$ type K from mutations in type I). $i + k - (a - x_i) = n - a - j + x_i$, giving $0 \le b \le n - a - j + x_i$.

The first restriction on x_j, the number (out of b) of type J mitochondria that mutate to type I, is $0 \le x_j \le b$. The second restriction is $x_j \le i$ (the number of mutations that go to type I cannot be larger than the number of required type I - type I has yet to receive any mutations from other types) and the third restriction is $b - x_j \le k - (a - x_i)$ (the number of mutations that go to type K must be less than or equal to the number of type K still needed by the cell), which when rearranged gives $x_j \ge i - n + a + b + j - x_i$. The intersection of these inequalities is $\max(0, i - n + a + b + j - x_i) \le x_j \le \min(i, b)$.

c, the number of type K mitochondria that mutate into type I or type K mitochondria, cannot be greater than the remaining complement of type I (now needs $i - x_j$ mitochondria after receiving x_j from type J), and J (still needs $j - x_i$ mitochondria). Thus, $c \le (i - x_j) + (j - x_i)$, which gives $0 \le c \le i + j - x_i - x_j$,

Finally, the first restriction on x_k, the number of c mutations in type K mitochondria that mutate to type I, is $0 \le x_k \le c$, the second restriction is $x_k \le i - x_j$, and the third restriction is $c - x_k \le j - x_i$, which can be rearranged to give $x_k \ge c - j + x_i$. The intersection of these inequalities gives $\max(0, c - j + x_i) \le x_k \le \min(i - x_j, c)$.

Once we have determined all valid combinations of mutation variables, we must determine the probability of each transition (a single combination of mutation variables) from a pre-mutation cell to our specified post-mutation cell. To determine the probability of a single transition, we multiply the probabilities associated with each mutation variable by the probability of the pre-mutation cell.

$Y(a; i + a - x_j - x_k, \mu) = Y(a; i', \mu)$ represents the probability of obtaining a mutations in pre-mutation cells that contain i' type I mitochondria. The accumulation of a mutations follows a binomial distribution,

$$Y(a; i', \mu) = \binom{i'}{a} \mu^a (1 - \mu)^{i - x_j - x_k},$$

where μ is the probability of a mitochondrion being chosen for mutation.

$Z(x_i; a, P_{ij})$ represents the probability that x_i mutations (out of a) become J mitochondria and $a - x_i$ mutations become K mitochondria, where each mutation becomes type J with probability P_{ij}. This follows a binomial distribution,

$$Z(x_i; a, P_{ij}) = \binom{a}{x_i} (P_{ij})^{x_i} (1 - P_{ij})^{a - x_i}. \qquad (9)$$

We let $Y\left(b; j+b-x_i-\left(c-x_k\right),\mu\right)=Y\left(b; j',\mu\right)$ represent the probability of obtaining b mutations in pre-mutation cells that contain j' type J mitochondria, where

$$Y(b; j', \mu) = \binom{j'}{b} \mu^b (1-\mu)^{j-x_i-(c-x_k)}.$$

Likewise, $Y(c; k+c-(a-x_i)-(b-x_j), \mu) = Y(c; k', \mu)$ is the probability of obtaining c mutations in the pre-mutation cells that contain k' type K mitochondria, where

$$Y(c; k', \mu) = \binom{k'}{c} \mu^c (1-\mu)^{k-(a-x_i)-(b-x_j)}.$$

$Z(x_j; b, P_{ji})$ is the probability that x_j (out of b) mutations become I mitochondria and $b-x_j$ mutations become K mitochondria, given that each mutation becomes type I with probability P_{ji}, where

$$Z(x_j; b, P_{ji}) = \binom{b}{x_j} (P_{ji})^{x_j} (1-P_{ji})^{b-x_j}. \qquad (10)$$

$Z(x_k; c, P_{ki})$ is the probability that x_k (out of c) mutations become I mitochondria and $b-x_j$ mutations become J mitochondria, where mutations become type I with probability P_{ki}, where

$$Z(x_k; c, P_{ki}) = \binom{c}{x_k} (P_{ki})^{x_k} (1 - P_{ki})^{c - x_k}. \tag{11}$$

When there are no fitness differences between the mitochondrial types, $P_{ij} = P_{ji} = P_{ki} = 0.5$ (i.e. there is no bias in mutation between I, J and K).

Putting everything together, we can determine the probability of any post-mutation cell via

$$P\left(\mathbf{M}^{t,\tau_3} = (i, j, G)\right) = \sum_{a=0}^{n-i} \sum_{x_i=\max(0, a-n+i+j)}^{\min(j,a)} \sum_{b=0}^{n-a-j+x_i} \sum_{x_j=\max(0, i-n+a+b+j-x_i)}^{\min(i,b)} \sum_{c=0}^{i+j-x_i-x_j} \sum_{x_k=\max(0, c-j+x_i)}^{\min(i-x_j, c)} \Big(Y(a; i', \mu)$$
$$\times Y(b; j', \mu) Y(c; k', \mu) Z(x_i; a, P_{ij}) Z(x_j; b, P_{ji}) Z(x_k; c, P_{ki}) P\left(\mathbf{M}^{t,\tau_2} = (i', j', G)\right)\Big).$$

We normalize the post-mutation population by

$$P\left(\mathbf{M}^{t,\tau_4} = (i, j, G)\right) = \frac{P\left(\mathbf{M}^{t,\tau_3} = (i, j, G)\right)}{\sigma},$$

where

$$\sigma = \sum_{i=0}^{n} \sum_{j=0}^{n-i} P\left(\mathbf{M}^{t,\tau_3} = (i, j, U_1 B_2)\right) + P\left(\mathbf{M}^{t,\tau_3} = (i, j, B_1 B_2)\right),$$

so that the sum of the proportions of the population equals 1.

Selection

The fitness function is substantially more complicated when we consider three mitochondrial types. We generated two fitness functions that are similar to the concave and convex fitness functions in the main model (Fig. S14). The fitness shape is given by

$$w'(i,j) = \left| \frac{e^{-tu}}{1+e^{-tu}} - 0.5 \right|, \tag{12}$$

where

$$u(i,j) = v\sqrt{(i-y)^2 + (j-y)^2 + (k-y)^2}\ .$$

t, v and y are variables that alter the shape and compression of the fitness function. The values of these variables were chosen to generate a three-dimensional equivalent to the concave fitness function and the convex fitness function (S29 Table and Fig. S14).

We normalize the fitness shape so that maximum fitness equals 1 and minimum fitness equals a pre-determined value, h (S29 Table). The cost of heteroplasmy, c_h, is given by $c_h = 1 - h$.

$$w(i,j) = \frac{(1-h)\left(w'(i,j) - \min(w')\right)}{\max(w') - \min(w')} + h,$$

where $\min(w')$ and $\max(w')$ are the minimum and maximum values of w' over the domain of valid values for i and j.

The state $\mathbf{M}^{t,\tau_5} = (i,j,G)$ represents cells after selection, which we determine via

$P\left(\mathbf{M}^{t,\tau_5} = (i,j,G)\right) = P\left(\mathbf{M}^{t,\tau_4} = (i,j,G)\right) w(i,j)$.

Meiosis

As in the main model, we sample n mitochondria with replacement from a cell containing n mitochondria and add the set of sampled mitochondria to the original set of mitochondria to form a cell containing $2n$ mitochondria. We let $\mathbf{M}^{t,\tau_6} = (l, m, 2G)$ represent the cell with doubled mitochondria and nuclear genotype, where l takes values in $\{0,1...2n\}$, m takes values in $\{0,1...2n-l\}$ and $2G$ takes values in $\{U_1U_1B_2B_2, B_1B_1B_2B_2\}$.

We denote the probability of sampling $l-i$ type I mitochondria and $m-j$ type J mitochondria from $\mathbf{M}^{t,\tau_5} = (i, j, G)$ as $F\left(l-i, m-j; n, \frac{i}{n}, \frac{j}{n}\right)$. Sampling follows a multinomial distribution, giving

$$F\left(l-i, m-j; n, \frac{i}{n}, \frac{j}{n}\right) = \frac{n!}{(l-i)!(m-j)!(n-(l-i)-(m-j))!}\left(\frac{i}{n}\right)^{l-i}\left(\frac{j}{n}\right)^{m-j}\left(\frac{k}{n}\right)^{n-(l-i)-(m-j)}.$$

We obtain $\mathbf{M}^{t,\tau_6} = (l, m, 2G)$ by

$$P\left(\mathbf{M}^{t,\tau_6} = (l, m, 2G)\right) = \sum_{i=\max(0,l-n)}^{\min(l,n)} \sum_{j=\max(0,m+l-n-i)}^{\min(m,n-i)} F\left(l-i, m-j; n, \frac{i}{n}, \frac{j}{n}\right) P\left(\mathbf{M}^{t,\tau_5} = (i, j, G)\right).$$

During the second step of meiosis, the cells with $2n$ mitochondria produce gametes with $n/2$ mitochondria. We define $S\left(p, q; 2n, l, m, \frac{n}{2}\right)$ to be the probability of obtaining p type I and q type J mitochondria in $n/2$ draws from the $\mathbf{M}^{t,\tau_6} = (l, m, 2G)$ cell that contains l type I and m type J mitochondria (out of $2n$ total mitochondria). Here, sampling is without replacement and follows a multivariate hypergeometric distribution, giving

$$S\left(p,q;2n,l,m,\frac{n}{2}\right)=\frac{\binom{l}{p}\binom{m}{q}\binom{2n-l-m}{\frac{n}{2}-p-q}}{\binom{2n}{\frac{n}{2}}}.$$

Gametes produced by meiosis are represented by $\mathbf{M}^{t+1,\tau_1}=(p,q,g)$. We determine the probability of obtaining a particular gamete using

$$P\left(\mathbf{M}^{t+1,\tau_1}=(p,q,U_1)\right)=\frac{1}{2}\left(\sum_{l=0}^{2n}\sum_{m=0}^{2n-l}S\left(p,q;2n,l,m,\frac{n}{2}\right)P\left(\mathbf{M}^{t,\tau_6}=(l,m,U_1U_1B_2B_2)\right)\right),$$

$$P\left(\mathbf{M}^{t+1,\tau_1}=(p,q,B_1)\right)=\frac{1}{2}\left(\sum_{l=0}^{2n}\sum_{m=0}^{2n-l}S\left(p,q;2n,l,m,\frac{n}{2}\right)P\left(\mathbf{M}^{t,\tau_6}=(l,m,B_1B_1B_2B_2)\right)\right),$$

and

$$\begin{aligned}&P\left(\mathbf{M}^{t+1,\tau_1}=(p,q,B_2)\right)\\&=\frac{1}{2}\left(\sum_{l=0}^{2n}\sum_{m=0}^{2n-l}S\left(p,q;2n,l,m,\frac{n}{2}\right)P\left(\mathbf{M}^{t,\tau_6}=(l,m,U_1U_1B_2B_2)\right)\right)\\&+\frac{1}{2}\left(\sum_{l=0}^{2n}\sum_{m=0}^{2n-l}S\left(p,q;2n,l,m,\frac{n}{2}\right)P\left(\mathbf{M}^{t,\tau_6}=(l,m,B_1B_1B_2B_2)\right)\right).\end{aligned}$$

Deleterious mutations

We alter the fitness function slightly to account for non-neutral mutations. First, we determine w' using equation (12) as before. We reduce the fitness of types I and J mitochondria via

$$w''(i,j) = w'(i,j)\left(1-\left(\frac{i\phi}{n}\right)^2\right)\left(1-\left(\frac{j\phi}{n}\right)^2\right),$$

where ϕ controls the severity of the deleterious mutation (S30 Table; column 1).

Fitness is normalized as before by

$$w_d(i,j) = \frac{(1-h)(w''(i,j)-w''(\min))}{w''(\max)-w''(\min)} + h.$$

We choose a value of ϕ that leads to cells that are homoplasmic for type I or J having a fitness of $1-s_d$ (i.e. $w_d(n,0) = w_d(0,n) = 1-s_d$).

We also alter equations (9), (10) and (11). Under the deleterious scenario, $P_{ij} = 0.99, P_{ji} = 0.99$ and $P_{ki} = 0.01$. The probability that a deleterious type mutates to a neutral type is lower than the probability that a deleterious type mutates to another deleterious type (as the former is effectively an advantageous mutation).

Advantageous mutations

Again, we determine w' using equation (12) but now type I mitochondria have an advantage, determined via

$$w''(i,j) = w'(i,j)\left(1+\left(\frac{i\varphi}{n}\right)^2\right),$$

where φ scales the benefit of the advantageous mutation (S30 Table; column 2). Fitness is normalized so that maximum fitness is 1,

$$w_a(i,j) = \frac{(1-h)(w''(i,j) - w''(\min))}{w''(\max) - w''(\min)} + h.$$

We choose a value of φ so that cells homoplasmic for type I have a fitness of 1, while cells homoplasmic for type J and K have a fitness of $1-s_a$ (i.e. $w_a(n,0)=1$ and $w_a(0,0) = w_a(0,n) = 1-s_a$).

Again, we also alter equations (9), (10) and (11). Under the advantageous scenario, $P_{ij} = 0.5, P_{ji} = 0.01$ and $P_{ki} = 0.01$. This accounts for the fact that advantageous mutations are less common than deleterious mutations (i.e. mutations from advantageous to neutral).
