# Supplementary figures and images for: Selection against Heteroplasmy Explains the Evolution of Uniparental Inheritance of Mitochondria
Source: PLoS Genet. 2015 Apr 16;11(4):e1005112. doi: 10.1371/journal.pgen.1005112 (PMC4400020; doi:10.1371/journal.pgen.1005112)

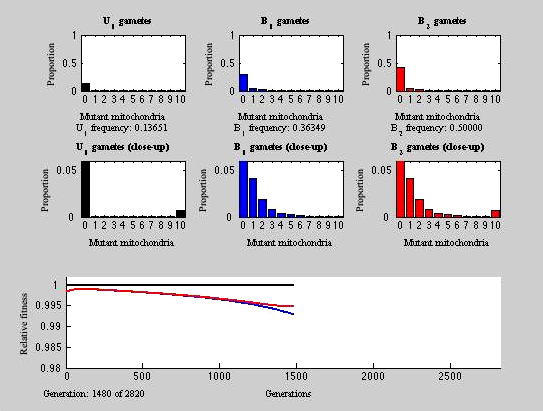

Supplement: S1 Video Still Image — (TIFF) [file pgen.1005112.s055.tiff]

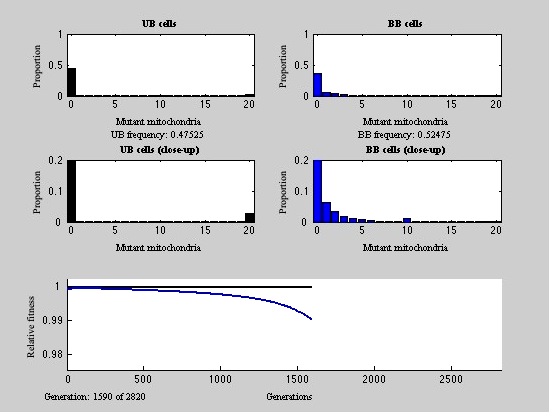

Supplement: S2 Video Still Image — (TIFF) [file pgen.1005112.s056.tiff]
